# Simultaneous two-color imaging with a dual-channel miniscope in freely behaving mice

**DOI:** 10.1126/sciadv.adr6470

**Published:** 2025-07-02

**Authors:** Zhe Dong, Yu Feng, Keziah Diego, Austin M. Baggetta, Brian M. Sweis, Zachary T. Pennington, Sophia I. Lamsifer, Yosif Zaki, Federico Sangiuliano, Paul A. Philipsberg, Denisse Morales-Rodriguez, Daniel Kircher, Paul Slesinger, Tristan Shuman, Daniel Aharoni, Denise J. Cai

**Affiliations:** ^1^ Nash Family Department of Neuroscience, Icahn School of Medicine at Mount Sinai, New York, NY, USA.; ^2^ Department of Psychiatry, Icahn School of Medicine at Mount Sinai, New York, NY, USA.; ^3^ Department of Neurology, David Geffen School of Medicine, University of California, Los Angeles, Los Angeles, CA, USA.

## Abstract

Miniaturized fluorescence microscopes (miniscopes) enable imaging of calcium events from a large population of neurons in freely behaving animals. Traditionally, miniscopes have only been able to record from a single fluorescence wavelength. Here, we present an open-source dual-channel miniscope that simultaneously records two wavelengths in freely behaving animals. To enable simultaneous acquisition of two fluorescent wavelengths, we incorporated two CMOS sensors into a single miniscope. To validate our dual-channel miniscope, we imaged hippocampal CA1 region that co-expressed a dynamic calcium indicator (GCaMP) and a static nuclear signal (dTomato) while mice ran on a linear track. Our results suggest that, even when neurons were registered across days using dTomato signals, hippocampal spatial coding changes over time. In conclusion, our dual-channel miniscope enables imaging of two fluorescence wavelengths with minimal cross-talk between the two channels, opening the doors to a multitude of previously inaccessible experimental possibilities.

## Teaser

An open-source dual-channel miniscope that simultaneously records two wavelengths in freely behaving animals was presented.

## INTRODUCTION

Recent years have seen rapid growth in the development and dissemination of open-source miniature fluorescence microscopes (miniscopes) (*1*–*3*). The miniscope allows circuit investigation of large populations of neurons across days in small animals such as rodents (*4*–*11*), birds (*12*), and bats (*13*). Because miniscopes are lightweight enough to be carried by animals while freely behaving, a broad range of rich behaviors can be studied including song vocalization (*12*), social interaction (*6*, *10*), spatial navigation (*4*), and memory integration and updating (*5*). The open-source UCLA Miniscope project is an impactful open-source initiative in the neuroscience community, with more than 700 laboratories building and using miniscopes to carry out independent research (*1*, *2*). Since its initial release, the UCLA Miniscope has iterated through several versions. The latest version of the UCLA Miniscope (v4) features a highly sensitive PYTHON480 complementary metal-oxide semiconductor (CMOS) sensor, a large 1 mm–by–1 mm field of view, fully achromatic optics, and electrowetting focus adjustment of ±200 μm (https://github.com/Aharoni-Lab/Miniscope-v4) (*5*). The open-source nature of the UCLA Miniscope also facilitates customization and innovations on top of the original design. Several alternative designs have improved imaging sensitivity and provided a more compact microscope size, larger field of view, and lighter weight, which collectively enables imaging across multiple brain regions and better overall quality (*14*–*20*). In addition, wireless versions of the miniscope allow animals to travel in a large environment and enable calcium imaging in behaving songbirds and bats (*11*, *21*). Furthermore, three-dimensional (3D) imaging using miniscopes is possible with both computational imaging and traditional optical imaging techniques (*22*–*25*). In summary, with these recent technological advancements, it is prime time to innovate on top of the latest hardware from the UCLA Miniscope project and further expand the functionality of miniscopes to pursue unanswered research questions.

One of the most common use cases for miniscopes is to image genetically encoded calcium indicators like GCaMP, which allows measurement of neural population activity at single-cell resolution (*2*). However, with the advancement of new genetic tools, it is beneficial to combine two or more fluorescent reporters to answer questions about the nervous system in unprecedented ways. There is a growing need to image two or more fluorescent reporters simultaneously at single-cell resolution. Multiple scenarios could benefit from dual-channel imaging. For example, one can image a GCaMP signal together with a static neuronal marker in the other channel. The static neuronal signal can improve motion correction within a recording session, image registration across recording sessions, and identification of neurons that are inactive during the recording. In addition, by using cell-type or activity-dependent markers, researchers may use the additional channel to differentiate cell types and characterize differences in neural activity specific to the genetic and activation profiles of neural ensembles (e.g., a static neuronal marker might identify GABAergic neurons while GCaMP is used to record from all neurons). Similarly, simultaneous imaging of two dynamic fluorescence signals across the two channels could shine light on how different neural populations coordinate and function together. For example, one may use GCaMP and RCaMP (a red-shifted calcium indicator) to mark excitatory and inhibitory neurons, respectively, and subsequently study the population dynamics of these two distinct populations. In summary, developing a miniscope that is capable of dual-channel imaging in behaving animals will fill a substantial gap in current neural imaging technologies.

One important application for a dual-channel miniscope is to use the additional channel to image a static neuronal marker labeling all neurons in the field of view, which allows researchers to track a neural population over time. Despite recent developments in registration algorithms (*26*), tracking the same population of cells across long periods of time (e.g., weeks to months) remains a challenge in calcium imaging, due to the dynamic nature of GCaMP signals (i.e., not all cells are active on a given recording session). The ability to track a neural population over time is particularly important when studying the stability of neural ensembles (e.g., hippocampal spatial representations). Recently, it has been reported that even when animals performed the same spatial navigation task in the same environment, the hippocampal spatial representation changed over time (i.e., representational drift) (*4*, *27*). This change is often described as a decrease of spatial representation stability (such as reactivation rate of place cells and population vector correlation) over time. However, because the imaging field of view may slightly change across days in behaving animals, it was previously unclear how much the instability of the field of view contributed to the instabilities in reactivation rate and population vector correlation. For instance, if a cell that appears in one session lies near the edge of the field of view (in the *xy* plane or the *z* direction), imaging this same cell in a subsequent session with a slightly changed field of view may result in a substantial change in signal level because the cell may no longer be in focus or may be completely outside the field of view. In other words, instability in the field of view alone could result in a “false” change in reactivation rate or population vector correlation that should not be attributed to biological changes in neural activity patterns. As a result, researchers often have to fall back to more conservative heuristics when measuring the change in hippocampal spatial representation. One common approach is to include only the neurons that can be registered using GCaMP signal throughout the experiment, which limits the analysis to the population of neurons that were always active over time, yielding a partial view of how the hippocampal spatial representation changes. In contrast, collecting a static landmark signal for each cell together with the GCaMP signal would allow us to detect and register cells even when they were not active during a recording session. Hence, imaging a static neuronal marker along with dynamic GCaMP signal is a prominent application of a dual-channel miniscope and enables researchers to study hippocampal spatial representation stability in an unprecedented way.

Here, we present a miniscope that is capable of dual-channel calcium imaging in behaving animals. Our design was derived from version 4 of the UCLA Miniscope with two high-sensitivity PYTHON480 CMOS sensors, 1 mm by 1 mm field of view, and electrowetting focus adjustment. The resolution of our dual-channel miniscope is 3.5 μm and all optics within the scope are achromatic (same as Miniscope v4). Our design includes a sliding mechanism to correct any displacement in the focal plane across the two channels caused by chromatic aberration when using a gradient refractive index (GRIN) lens to image deeper brain regions. The fully assembled dual-channel miniscope weighs 4.8 g and can be carried by freely behaving mice. Table 1 summarizes key specifications and features of the presented dual-channel miniscope together with comparable miniature microscopes that are now available. To validate imaging of two wavelengths of light in freely behaving animals and to demonstrate the application of the dual-channel miniscope in the study of hippocampal spatial representations, we performed an imaging experiment where animals ran along a linear track across days. We injected a virus which co-expressed GCaMP and a constitutively active dTomato in the CA1 region of the hippocampus. The constitutively active dTomato signals provided a stable marker for cells that were within the imaging field of view. Our results are consistent with prior reports, suggesting a change in the hippocampal spatial representation over time even when the instability of field of view is accounted for using the additional dTomato channel, demonstrating a prominent application of our dual-channel miniscope.

**Table 1.** A selection of now available miniature microscopes. Open-source and commercial miniature microscopes are shown together with their key specifications and features. *L*, length; *W*, width; *H*, height; *D*, depth; FOV, field of view; fps, frames per second; NA, specifications not available or not applicable.

	Weight (g)	Size (*L* by *W* by *H*, mm)	Resolution (*W* by *H*, pixels)	FOV (*W* by *H*, μm)	Frame rate (fps)	Number of channels	Connection	Remarks
Dual-channel miniscope	4.8	13.96 by 11.32 by 31.55	608 by 608 (each channel)	1000 by 1000 (each channel)	30 (each channel)	2	Wired	Presented work
CHEndoscope	4.5	NA	NA	NA	NA	1	Wired	Open-source (*14*)
Doric Lenses 1-color	2.2	13.9 by 8.8 by 16.6	630 by 630	700 by 700	49	1	Wired	Commercial solution (*43*)
Doric Lenses 2-color	3.7	17 by 9.5 by 18	600 by 600	320 by 320	45	2	Wired	Commercial solution
Featherscope	1.0	NA	608 by 608 (max)	1000 by 1000	30 (max)	1	Wired	Open source (*18*)
FHIRM-TPM 2.0	4.2	16 by 9 by 30	512 by 512	420 by 420	10	1	Wired	Open source; miniature two-photon imaging design (*44*)
FinchScope	3.8	NA	640 by 480	800 by 600	30	1	Wireless	Open source; compact, wireless design suitable for imaging in song birds and bats (*21*)
Inscopix nVista	2.2	15 by 8.8 by 22	1280 by 800	1050 by 650	60	1	Wired	Commercial solution (*45*)
Inscopix nVue	2.4	14.8 by 8.5 by 21.5	1280 by 800	1050 by 650	30	2	Wired	Commercial solution (*30*)
MINI2p	2.4	NA	256 by 256	420 by 420	40	1	Wired	Open source; miniature two-photon imaging design (*46*)
MiniFast	3.45	22 by 13 by 21–25	1920 by 1080 (max)	NA	500 (max)	1	Wired	Open source; high sampling rate design (*17*)
MiniLFM	4.71	NA	1280 by 1024	700 by 600 by 360 (*W* by *H* by *D*, μm)	16	1	Wired	Open source; volumetric calcium imaging design (*22*)
Miniscope 2P	4	NA	NA	445 by 380	NA	2	Wired	Open source; miniature two-photon imaging design (*47*)
Miniscope LFOV	13.9	35 by 20 by 30	1296 by 972	3600 by 2700	23	1	Wired	Open source; larger field-of-view design (*20*)
Miniscope v4	2.6	17.52 by 15.63 by 22.42	608 by 608	1000 by 1000	30	1	Wired	Open source (*2*, *5*)
Miniscope Wire-free	4.7	18.5 by 17.05 by 27.24	320 by 320	700 by 450	20	1	Wireless	Open source; wire-free design (*11*)
NINscope	1.6	11 by 11 by 18.2	752 by 480	786 by 502	30	1 (up to 2)	Wired	Open source; compact design suitable for simultaneous dual-region recording (*15*)
SIMscope3D	6.65	22.91 by 22.91 by 29.06	NA	207 by 207	NA	1	Wired	Open source; volumetric calcium imaging design (*48*)

## RESULTS

### Dual-channel miniscope design

**Fig. 1.**
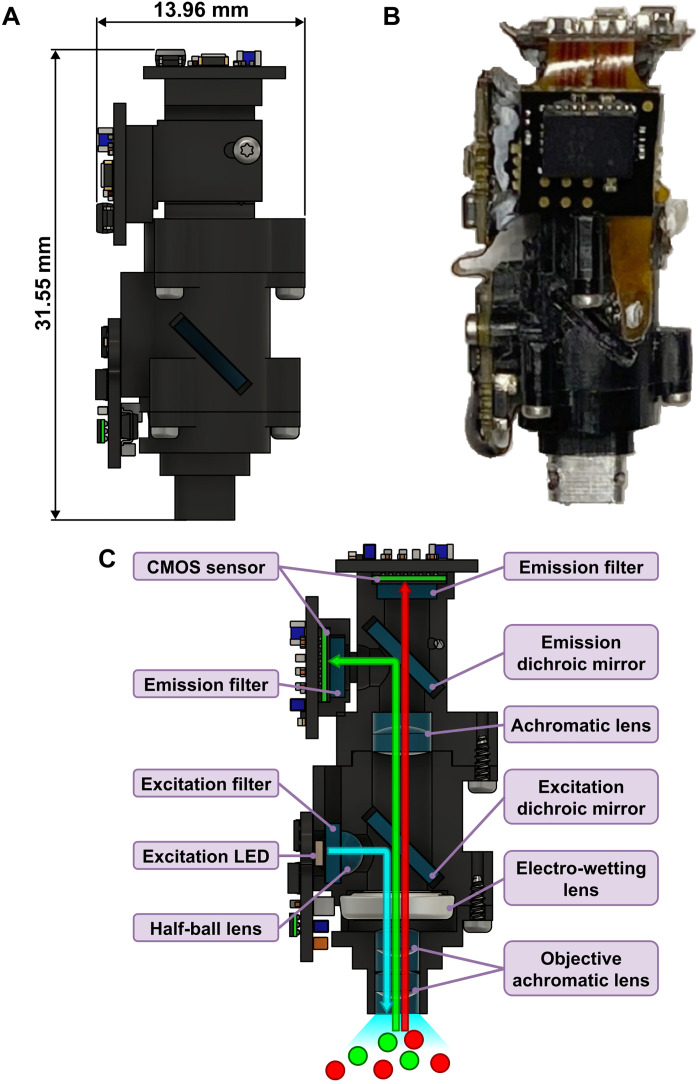
Dual-channel miniscope design. (**A**) 3D rendering of the dual-channel miniscope from the side showing the height (31.55 mm) and diameter (13.96 mm) of the miniscope body. (**B**) Photo of the dual-channel miniscope from the side. (**C**) Cross section of the dual-channel miniscope showing the components and light path. The LED provides excitation light (cyan arrow). The excitation light travels through excitation filter and half-ball lens and then gets deflected by the dual-band excitation dichroic mirror, followed by the electro-wetting lens and objective achromatic lens stack. Once the sample is illuminated, the emission light with two wavelengths (green and red arrows) travels straight up into the dual-channel miniscope. The two wavelengths pass through the objective achromatic stack, the electro-wetting lens, and the excitation dichroic mirror and are focused by the achromatic lens. The wavelengths are then split by the high-pass emission dichroic mirror, which deflects the lower wavelength (green) and passes through the higher wavelength (red). Last, emission light is collected independently with two CMOS sensors with corresponding single–band-pass emission filters.

We built our proof-of-concept dual-channel miniscope to image GCaMP and dTomato, but it could be used to image any two fluorophores by adjusting the appropriate filters and light-emitting diode (LED). Figure 1 shows a schematic of the design. To achieve simultaneous dual-channel imaging, we used a dichroic mirror to split the two emission wavelengths and used two CMOS sensors to acquire the images from two channels simultaneously. The excitation LED provides the light source for excitation. Depending on the specific application, different types of excitation LED can be installed that can either emit white (RGB), dual wavelengths, or even single-wavelength excitation light, as long as it provides enough power to excite both fluorophores. Here, we used an LED with a single cyan wavelength that is strong enough to excite both GCaMP and dTomato. The excitation light then travels through a dual–band-pass excitation filter that selectively passes through the two excitation wavelengths, followed by a half-ball lens to provide filtered and uniform excitation light. Next, the dual-band excitation dichroic designed to specifically reflect the two excitation wavelengths reflects the excitation light toward the sample. The excitation light then passes through the electrowetting lens followed by an objective achromatic lens stack and is focused onto the sample. The emission light (containing two wavelengths) travels through the achromatic lens stack and becomes collimated. By passing the excitation and emission lights through the same electrowetting lens and achromatic stack, we ensure that the focal plane is the same for excitation and emission and can be adjusted together using the electrowetting lens. The emission light then travels through the electrowetting lens and the dual-band excitation dichroic and is refocused by the emission achromatic lens. The two wavelengths in the emission light are split by a high-pass emission dichroic. The higher wavelength passes through the emission dichroic and is collected by the CMOS sensor on the top, while the lower wavelength is reflected by the emission dichroic and collected by the CMOS sensor on the side. An emission filter is placed before each CMOS sensor to pass through the specific emission wavelength and minimize cross-talk between the two channels. Because all of the optics in the dual-channel miniscope are achromatic, we placed the CMOS sensor in a way so that the distances traveled by both emission wavelengths are equal from the emission achromatic lens to the CMOS sensor, and the distance matches the focal length of the emission achromatic lens. This ensures that the focal plane is the same across both channels. Because the imaging principle is the same across different fluorophores with different excitation and emission wavelengths, our design is versatile to image a range of different combinations of fluorophores. The only modification needed to image a different set of fluorophores is to replace the excitation LED, excitation/emission filters, and dichroics to match the specific excitation/emission wavelengths of the fluorophores. Table S1 summarizes the bill of materials needed to assemble the dual-channel miniscope, with the LED and filter set designed to image dynamic GCaMP signals and static dTomato signals simultaneously. This configuration was used in the benchmarking and in vivo validation experiments described below.

**Fig. 2.**
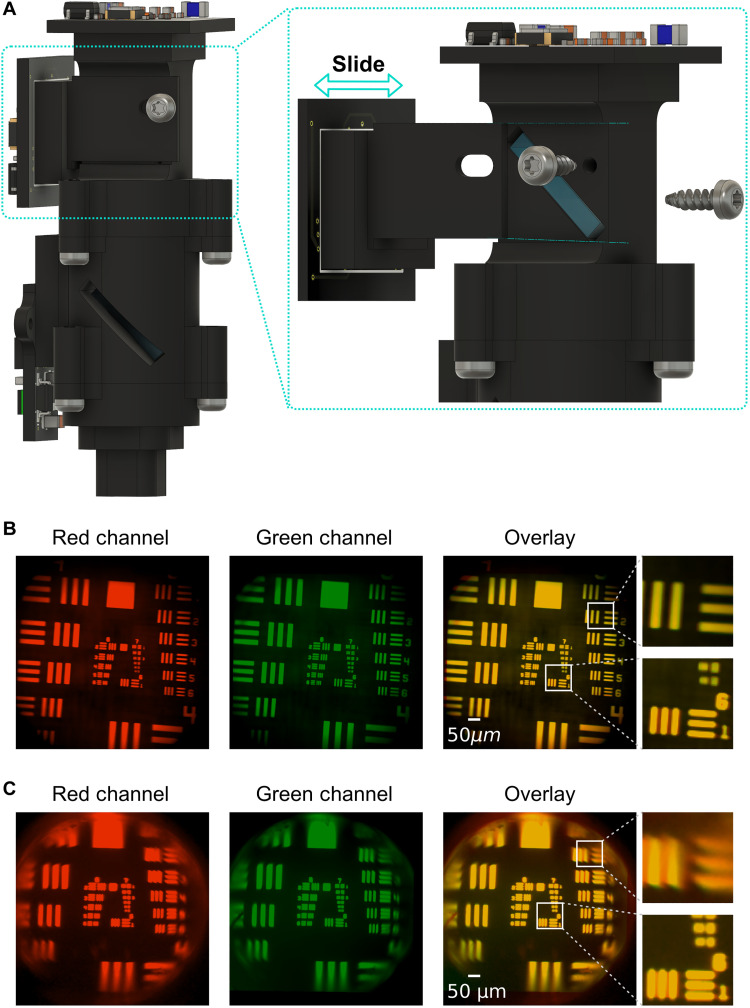
Sliding mechanism of the dual-channel miniscope. (**A**) 3D rendering of the dual-channel miniscope. The inset shows how the side CMOS sensor can slide along the optical path and be secured with two screws, which allows calibration of focal plane after assembly. (**B**) Image of a 1951 USAF resolution test target collected from red channel (top sensor) and green channel (side sensor) without a GRIN lens. An overlay of the two images demonstrates the same field of view across the two channels. The two zoomed-in insets show overlay at the center and edge of the field of view. (**C**) As in (B), with the addition of a 0.5-pitch 1-mm-diameter GRIN lens between the scope and the test target. Deformation caused by the GRIN lens is visible near the edges of the images. The field of view is the same across the two channels despite chromatic aberration introduced by the GRIN lens.

Although the dual-channel miniscope is designed to be achromatic, in practice, a GRIN lens is often needed to relay and reproduce images from deeper brain regions to the working distance of the dual-channel miniscope, which introduces chromatic aberration and causes a relative displacement in the focal plane across the two channels. Because the actual displacement depends on the pitch and diameter of the GRIN lens used, it is important to be able to correct for this displacement after assembling the dual-channel miniscope. The design described here includes a sliding mechanism that allows the user to calibrate the focal plane of the two channels independently during a calibration imaging session (Fig. 2). The CMOS sensor on the side is attached to a modular piece that fits onto the main body of the scope (Fig. 2A). This modular piece is able to slide along the light path, changing the placement of the side CMOS sensor, which essentially changes the relative displacement between the focal planes across the two channels. The modular piece can be secured by two screws once the desired placement is determined, which remains stable throughout an imaging experiment. Using this mechanism, users can calibrate the focal planes of the two channels on the bench by imaging a test target through the specific GRIN lens used in the experiment. After calibration, we observed a high degree of overlap between the two channels both with and without a GRIN lens (Fig. 2B), which suggests that this calibration process ensures imaging of the same focal plane even when imaging through a GRIN lens (Fig. 2C).

### Validation of dual-channel miniscope in freely behaving animals

**Fig. 3.**
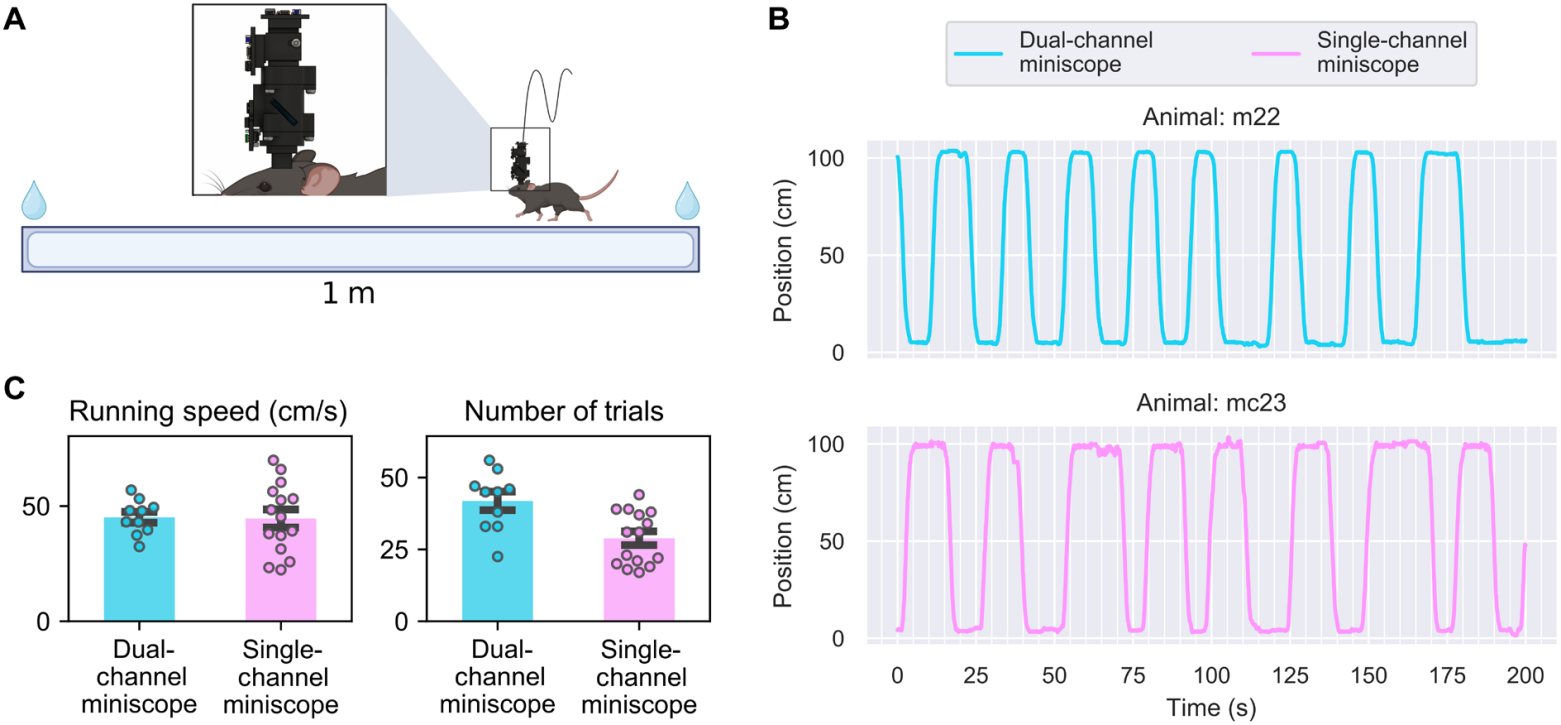
Imaging experiment using dual-channel miniscope. (**A**) Schematic of the imaging experiment. Mice run on a 1-m-long linear track to retrieve water rewards at both ends while wearing the dual-channel miniscope. (**B**) Locomotion of representative animal wearing dual-channel or single-channel miniscope while running on a 1-m-long linear track. (**C**) Comparison of running speed and number of trials between animals wearing single-channel miniscope and animals wearing dual-channel miniscope. The running speed was not significantly different between the two groups of animals. The number of trials for animals wearing dual-channel miniscope was significantly higher than animals wearing single-channel miniscope, likely due to extended training time in dual-channel miniscope animals.

To track the same population of cells across long periods of time and to validate that we are able to image the same focal plane across two channels in freely behaving animals, we injected a virus that co-expressed GCaMP6f together with a static dTomato in the same cell (fig. S1). We trained water-restricted mice to run on a linear track to retrieve 15 μl of water rewards at both ends of the track while imaging hippocampal CA1 region using our dual-channel miniscope (Fig. 3A and file S1). Mice performed well on the linear track while wearing the dual-channel miniscope (Fig. 3B), reaching speeds of 45.11 cm/s (±2.35 cm/s) when running (defined as 95th percentile of locomotion speed; see Materials and Methods) and ran an average of 41.43 (±3.37) trials during 15 min of recording. To investigate the behavioral impact of the dual-channel miniscope, we compared the running speed of animals in the dual-channel imaging experiment to another group of animals running on a linear track with a single-channel miniscope. The running speed of animals wearing dual-channel and single-channel miniscope was 45.11 and 44.61 cm/s, respectively, with no significant difference between the two groups ( P=0.924 , independent *t* test). The average number of trials with dual-channel miniscope was 41.85, significantly higher than 28.87 for single-channel miniscope ( P=0.003 , independent *t* test). Notably, to reach a comparable behavioral performance with the dual-channel miniscope, animals need to be habituated for longer (4 weeks instead of 1 to 2 weeks for single-channel miniscope). This is due to the increased weight of the dual-channel miniscope. The extensive training also explains the increased number of trials performed by animals wearing the dual-channel miniscope, where animals were much more familiar with the environment and could collect rewards faster. Additionally, to investigate the behavioral impact of the dual-channel miniscope in a more cognitively complex task, we tested mice wearing single-channel and dual-channel miniscopes in a neuroeconomic task called Restaurant Row (see Materials and Methods). We then analyzed key behavioral metrics, including total distance traveled, total valid laps (trials), and total reward earned in the Restaurant Row task. Our results showed that there was no significant difference in these measures when mice wore the dual-channel scope compared to the single-channel scope (fig. S2). Together, these results suggest that, given enough training time, the dual-channel miniscope has minimal behavioral impact for imaging experiments, similar to typical single-channel miniscopes.

**Fig. 4.**
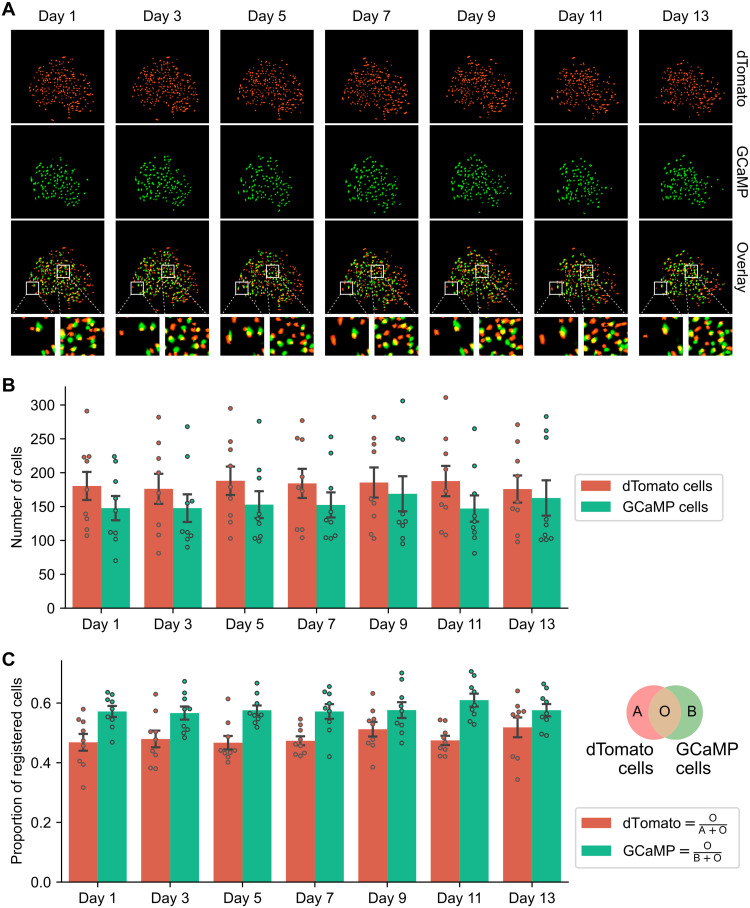
Cell registration across two channels. (**A**) Representative field of view from a single animal throughout the experiment. Processed spatial footprints are pseudo-colored and shown for the dTomato (top row) and GCaMP (middle row) channels and the overlay images (bottom row). The zoomed-in insets show the overlay at the center and edge of the field of view for each day. Cells from the GCaMP channel that cannot be registered to the dTomato channel are excluded from the plot. (**B**) Number of cells detected in each session for the dTomato and GCaMP channels. (**C**) Proportion of cells registered across two channels. The Venn diagram illustrates how the proportion was calculated for dTomato and GCaMP channel in each session. The numerator was the number of registered cells across the two channels. The denominator was the total number of dTomato and GCaMP cells for the dTomato and GCaMP channel, respectively.

To validate that we can track the same population of cells across a long period of time, we repeated the 15-min-long recording with 2-day intervals between sessions for a total of seven sessions, spanning 13 days. We then processed the GCaMP channel and dTomato channel independently to extract the spatial footprints of cells. Next, we extracted the temporal activity of the detected cells. For the GCaMP channel, we applied the CNMF algorithm to extract the calcium dynamics and refined the spatial footprints of each cell. For the dTomato channel, we projected raw fluorescence pixel values onto the spatial footprint of each cell. We then registered the footprint of each cell in the GCaMP channel to a footprint in the dTomato channel based on the distance between centroids. A representative field of view with extracted spatial footprints is shown in Fig. 4A. The maximum projection of the processed imaging data from the same field of view is shown in fig. S3. The distribution and shape of spatial footprints were highly similar across the two channels (Fig. 4A), suggesting that both channels are imaging the same field of view.

We then quantified the number of cells detected in each recording session, as well as the number of cells that were registered across the two channels, as a fraction of total number of cells in either the GCaMP or dTomato channel (Fig. 4, B and C). Each day, around 150 cells were detected in the field of view for each channel. Among these cells, ~50% of them can be registered, either as a fraction of GCaMP channel cell counts (Fig. 4C, green bars) or as dTomato cell counts (Fig. 4C, red bars). It is expected that not all dTomato cells can be registered to a GCaMP cell in each day because hippocampal neural activity is sparse. However, in an ideal case, all GCaMP cells should have a corresponding cell in the dTomato channel. Several factors might explain why the proportion of registered cells did not reach 100% for the GCaMP channel. First, we are using an excitation LED that is optimized for GCaMP but not dTomato (table S1), so it is possible that not all cells were visible in the dTomato channel each day. Second, different cells might have different relative expression levels for GCaMP and dTomato, which could result in cells that were visible in the GCaMP channel but not dTomato channel. Last, because we do not expect calcium dynamics in the dTomato channel, we cannot use calcium dynamic-based models to extracted cells from dTomato. Instead, we had to resort to approaches based purely on fluorescence intensity, which makes it more challenging to detect cells in the dTomato channel compared to the GCaMP channel. None of these factors affect our ability to interpret neural activity from GCaMP cells that can be registered with the dTomato channel.

**Fig. 5.**
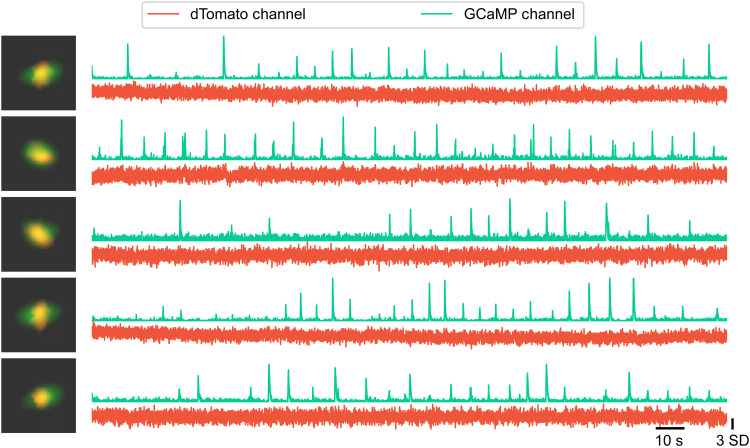
Representative cells registered across dTomato and GCaMP channels. The processed spatial footprint for each cell is pseudo-colored for the dTomato (red) and GCaMP (green) channels. A zoomed-in overlay image of the spatial footprint is shown for each cell on the left. The corresponding *z*-scored raw fluorescence from both channels is shown for each cell on the right. The raw fluorescence traces, obtained by projecting raw pixel values onto the highly overlapping spatial footprints, demonstrate minimal cross-talk between the channels.

To assess for cross-talk between channels, we investigated the cells registered across both channels and compared the corresponding temporal activity between the GCaMP and dTomato channels. There was no visible change in the fluorescence in the dTomato channel when a calcium event was visible in the GCaMP channel, despite the almost overlapping spatial footprints from the two channels, suggesting that there is minimal cross-talk from the GCaMP channel to the dTomato channel (Fig. 5).

**Fig. 6.**
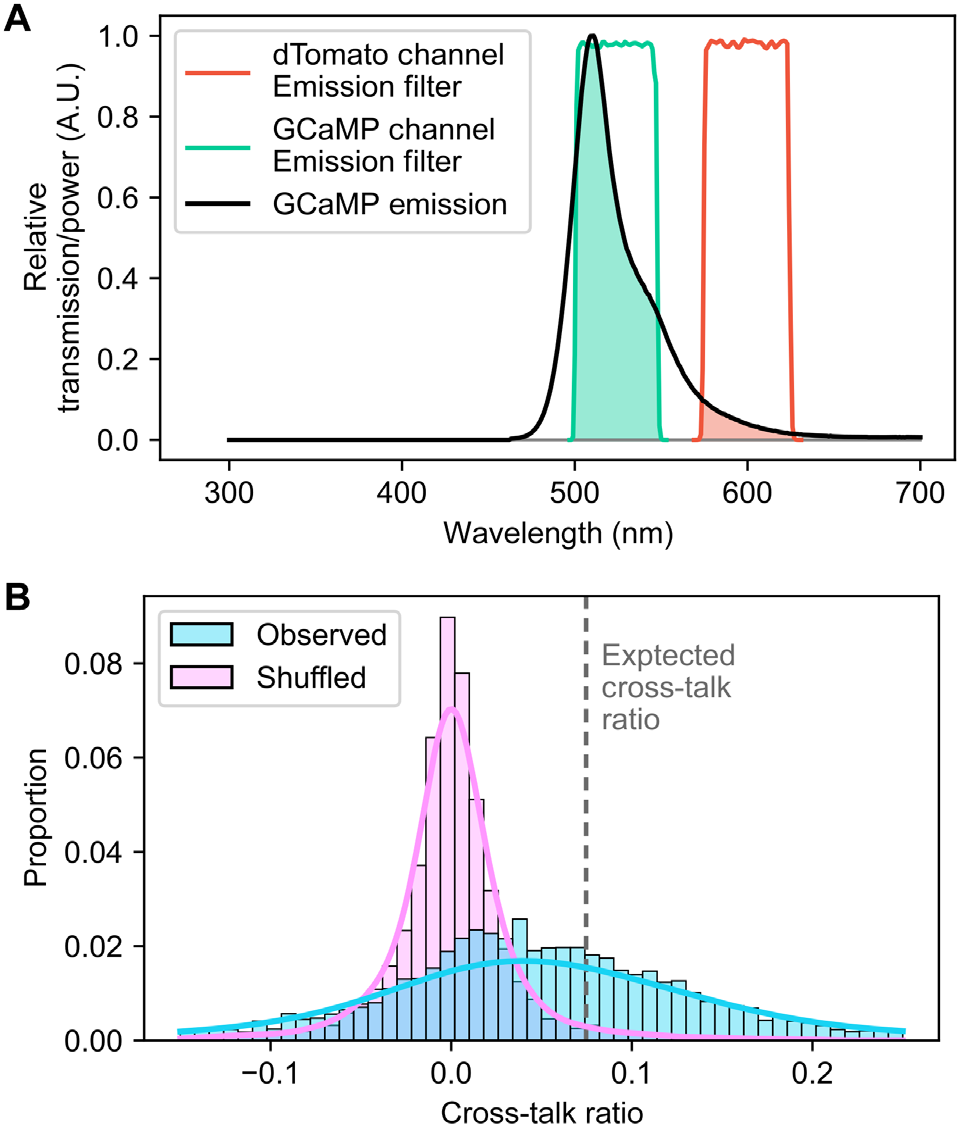
Quantification of cross-talk between the two channels. (**A**) Illustration of how the expected cross-talk ratio is calculated. The emission spectrum of GCaMP is shown in arbitrary units (A.U.), overlaid with the relative transmission profile for the GCaMP and dTomato channel emission filter. Green-shaded area indicates the relative amount of GCaMP emission that passes through the GCaMP channel filter; red-shaded area indicates emission passing through the dTomato channel filter. The expected cross-talk ratio is calculated as the ratio between the red and green areas. (**B**) Distribution of cross-talk ratio estimated from experimental data. The distribution of estimated cross-talk ratio (linear model coefficient) is shown in cyan, with the shuffled distribution overlaid in purple. The expected cross-talk ratio is also shown as a dashed line.

To quantify cross-talk, we calculated an expected cross-talk ratio from the GCaMP channel into the dTomato channel. The ratio is calculated as the amount of light in the GCaMP emission spectrum that passes through the dTomato channel emission filter, divided by the amount of light passing through the GCaMP channel emission filter (Fig. 6A). The resulting expected cross-talk ratio is 0.075 (i.e., in theory around 7.5% of fluorescence in GCaMP channel can be seen in the dTomato channel). We then estimated the same cross-talk ratio from experimental data that we collected. Leveraging the fact that the dTomato channel should not contain any calcium dynamic in the ideal setup, we estimate the cross-talk ratio by linearly fitting the temporal dynamic of the dTomato channel to that of the GCaMP channel for each cell registered across the two channels. Thus, the resulting linear model coefficient represented the amount of GCaMP dynamic that can be seen in the dTomato channel as a fraction of those seen in the GCaMP channel, providing an estimation of the observed cross-talk ratio. The distribution of estimated cross-talk ratio is shown in Fig. 6B. In addition, we generated a null distribution of the cross-talk ratio by randomly shuffling the temporal dynamic of one channel relative to the other for each cell registered across the two channels. The shuffled distribution centered around 0, representing no cross-talk (Fig. 6B). The mean of the observed cross-talk ratio was significantly different from both the mean of the shuffled distribution ( P<0.001 , paired *t* test) and the expected cross-talk ratio ( P<0.001 , one-sample *t* test). These results suggest that, although there is a nonzero amount of cross-talk, it is relatively minimal and less than the theoretical cross-talk ratio of 7.5%.

Together, these results suggest that using our dual-channel miniscope, we can image the same field of view across two channels with minimal cross-talk in freely behaving mice. Taking advantage of an additional static channel, we can track large population of cells in the hippocampal CA1 region across weeks.

### Investigation of hippocampal neural ensemble stability using the dual-channel miniscope

With the capability to track a large population of CA1 cells across weeks to months, we further investigated the stability of hippocampal spatial representations. The change in hippocampal spatial representations has been reported in two distinct ways. First, the reactivation rate of the neural population decreases as the time interval increases, suggesting that different populations of cells are active at different times (*4*, *27*). Second, the spatial population vector correlation decreases as the time interval increases, suggesting that the spatial representation is constantly changing over time (*4*, *27*). Both metrics can be influenced by instability of the field of view, potentially resulting in overestimation of the rate of changes over time. Hence, the instability of the field of view must be accounted for when studying the long-term stability of hippocampal spatial representations.

**Fig. 7.**
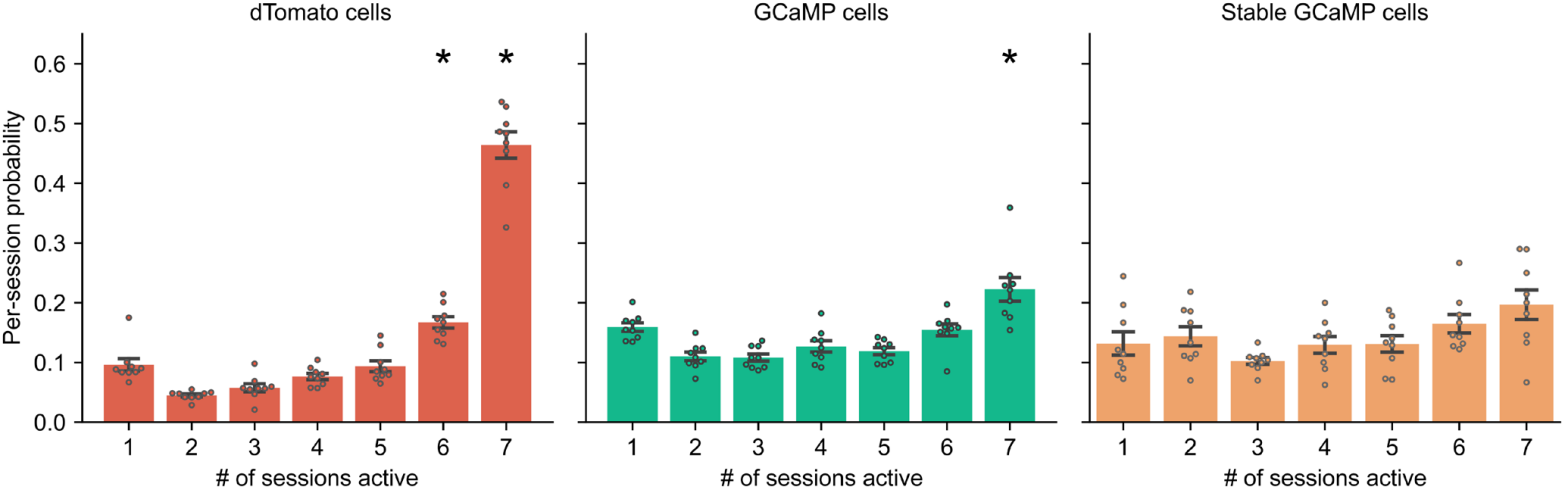
Tracking cells across weeks. Average per-session probabilities of cells tracked through different number of sessions are shown. The probability is estimated for each individual session as the number of cells tracked through certain number of sessions divided by the total number of cells in the session. The probability is then averaged across all sessions. The tracking result using the dTomato channel is shown on the left. The tracking result using only the GCaMP channel is shown in the middle. Then, we registered each GCaMP cell to a corresponding landmark dTomato cell, and aggregated only the GCaMP cells whose corresponding dTomato landmark can be tracked stably across all seven recording sessions. The tracking result of this stable subset of GCaMP cells is shown on the right. Star indicates a particular group (per-session probability for a particular number of sessions) was different from all other probabilities in post hoc pairwise Tukey’s post hoc tests.

To account for instability in the field of view, we used the additional static dTomato channel and studied the subset of cells that we can stably track across all 13 days of the imaging experiment. We investigated the stability of cells in each channel by quantifying (on a per-session basis) the probability of a cell being tracked in a different number of sessions (Fig. 7). A more detailed breakdown of proportion of cells activated at different combinations of sessions can be found in fig. S4. Overall, we were able to track a large proportion of cells through all seven recording sessions in the dTomato channel. In each session, on average, 46.4% of the cells were stably tracked in the dTomato channel throughout all seven imaging sessions. There was a significant group (number of sessions) effect [ $F_{6,56}=179.975,P<0.001$ , type 3 analysis of variance (ANOVA)], and the per-session probability of cells tracked across six and seven sessions was significantly different from the rest of probabilities (null hypothesis was rejected in all pairwise Tukey’s post hoc tests involving given group). Notably, not all cells in the dTomato channel can be tracked throughout all sessions, although dTomato expression is constitutive. This can be explained by the possibility that different cells might have different levels of dTomato expression through time or that not all cells in the field of view can be detected in the dTomato channel due to previously mentioned reasons. An alternative explanation is that there might be instability in the field of view, where cells may move in and out of the field of view, driving down the probability of active cells across multiple sessions, highlighting the need for a constitutively active cell marker. As a comparison, when registering cells using only the GCaMP channel, the probability of cells active across all seven sessions decreased to 22.2%. There was still a significant group (number of sessions) effect ( $F_{6,56}=15.212,P<0.001$ , type 3 ANOVA), and the per-session probability of cells tracked across seven sessions was significantly different from the rest of probabilities (null hypothesis was rejected in all pairwise Tukey’s post hoc tests involving a given group). It is not unexpected that a smaller proportion of cells were active across all seven recording sessions in the GCaMP channel. This is likely due to the sparsity of hippocampal neural activity, as not all cells are constantly active in any given session. However, it is still possible that instability of field of view may contribute to instability of neural activity across sessions. Because of this, we then focused our analysis on a subset of GCaMP cells, where the GCaMP cells can be registered to a stable subset of dTomato cells that can be tracked across all seven sessions. By extension, we call this subset of GCaMP cells the “stable GCaMP cells,” in the sense that we can be confident in their presence in the field of view, whether they were active or not, despite potential instability of field of view. In the stable GCaMP cells, on average, only 19.7% of these cells were active across all seven sessions, which is lower than the probability from all GCaMP cells. There was still a significant group (number of sessions) effect ( $F_{6,56}=3.335,P=0.007$ , type 3 ANOVA), but the probability of cells tracked across seven sessions was not significantly different from all other probabilities (pairwise Tukey’s post hoc test). Instability of field of view can no longer account for this result because the cells are all registered with dTomato channel that ensures that they are still present in the field of view across all sessions. Together, these results suggest that hippocampal neural activity was sparse and that different cells were active at different times even when instability of the field of view was accounted for.

**Fig. 8.**
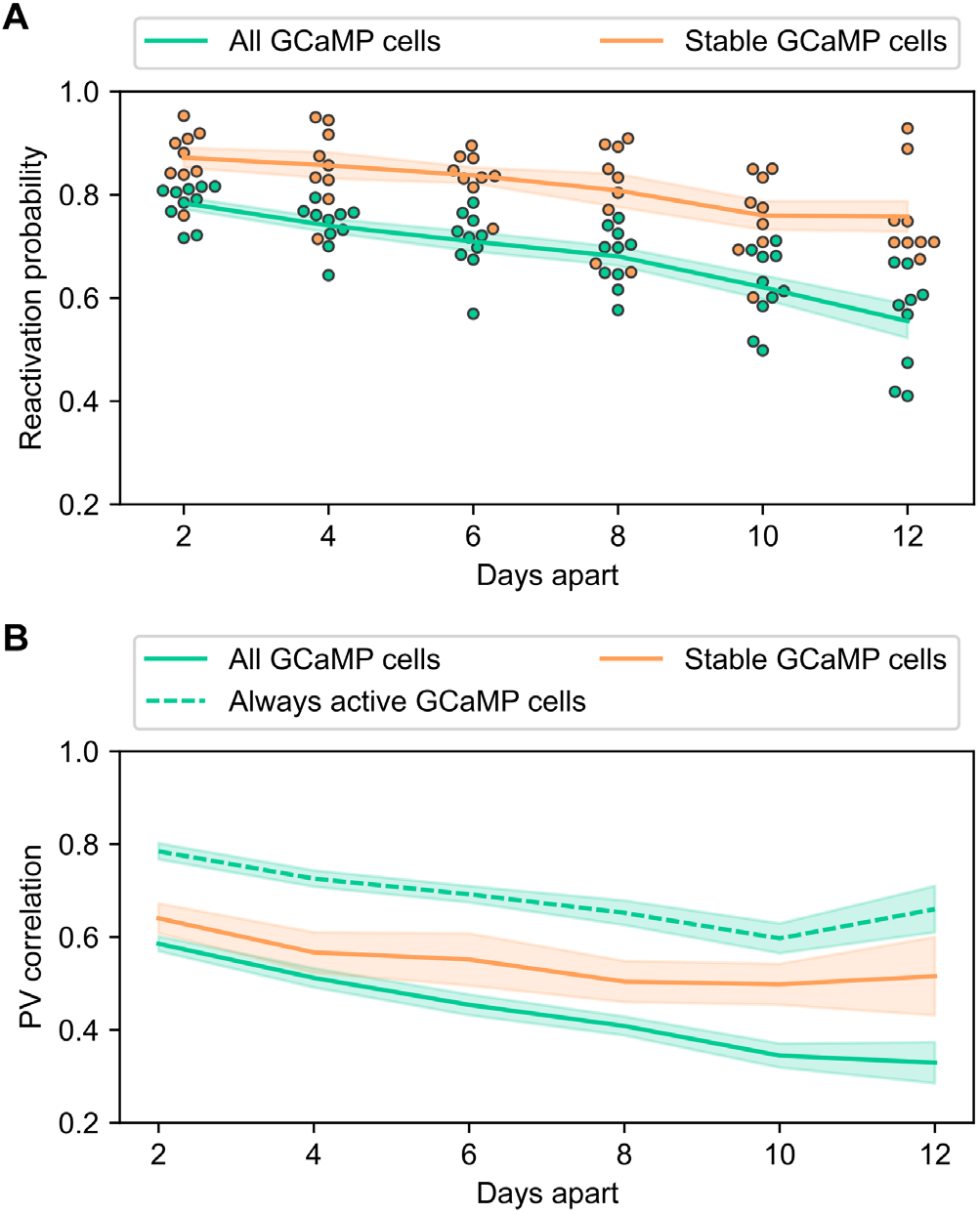
Hippocampal spatial representation changes over time. (**A**) Reactivation probability of cells across pairs of sessions as a function of time interval. The reactivation probability of cells for a pair of sessions is calculated as the number of cells shared across both sessions divided by the total number of cells in either session, then averaged across the pair of sessions. Solid lines show the mean across all possible combination of sessions with certain time interval and all animals, and shaded area shows the SEM. Green: All cells in GCaMP channel are included. Orange: Only GCaMP cells whose corresponding dTomato landmark can be tracked stably across all sessions are included. (**B**) Population vector correlation across pairs of sessions as a function of time interval. The population vector correlation is calculated as the mean Pearson correlation of population vectors across all spatial bins between a pair of sessions. Then, a mean is taken within each animal for a given time interval across all possible combinations of recording session pairs. Lines show the mean across all animals and shaded area shows the SEM. This calculation is carried out across all GCaMP cells (green solid line) and cells whose corresponding dTomato landmark can be tracked stably across all sessions (orange solid line). As a comparison, the same calculation is carried out with GCaMP cells that were active in both sessions in a pair regardless of the corresponding dTomato landmarks (green dashed line).

Next, we focused our analysis on place cells (*28*, *29*) (see definition of place cells in Materials and Methods) and investigated the stability of the hippocampal neural ensembles as a function of time interval. As shown in Fig. 8A, the reactivation probability of cells across pairs of sessions decreased as a function of time interval when we include all GCaMP cells [slope of −0.0217, P<0.001 , ordinary least squares (OLS)]. This result is consistent with previous findings on long-term stability of hippocampal spatial representation (*4*). As a comparison, when we only included the stable GCaMP population, the reactivation rate was much higher compared to the average of all cells registered with the GCaMP channel. As expected, the reactivation rate still decreased as a function of time for cells registered with dTomato but to a lesser degree (slope of −0.0127, P<0.001 , OLS). Overall, there were significant effects of time interval ( $F_{1,109}=67.74,P<0.001$ , type 3 ANOVA), cell inclusion ( $F_{1,109}=5.72,P=0.019$ , type 3 ANOVA), and the interaction between the two factors ( $F_{1,109}=5.64,P<0.019$ , type 3 ANOVA). We expected that reactivation probability would be lower when including all GCaMP cells because including cells that cannot be stably tracked essentially increases the denominator during the calculation of probability, yielding a numerically smaller result. Together, these results suggest that, consistent with previous findings, the reactivation probability of cells decreases as a function of time interval, even when the instability of field of view is accounted for using static landmarks in the dTomato channel.

After examining the reactivation of hippocampal neural ensembles, we proceeded to specifically investigate the stability of hippocampal spatial representations. As shown in fig. S5, within each day, the hippocampal place cell ensemble formed a spatial representation of the linear track, but the representation was not stable across days. This pattern was consistent regardless of whether we included all place cells found in the GCaMP channel or if we included only the place cells in the stable GCaMP population. We then calculated the spatial population vector correlation across days using place cells (Fig. 8B) with different cell inclusion criteria based on the two channels. Either all GCaMP cells (Fig. 8B, green solid line) or the stable GCaMP population (Fig. 8B, orange solid line) was considered. As an additional comparison, we also carried out the same analysis for GCaMP cells that were active during both sessions in the pairwise calculation regardless of the corresponding dTomato landmarks. As shown in Fig. 8B, the population vector correlation decreased as the time interval increased for all three inclusion criteria (all GCaMP cells: slope of −0.026, P<0.001 ; stable GCaMP cells: slope of −0.013, P=0.048 ; and always active GCaMP cells: slope of −0.015, P<0.001 , OLS). Overall, there was a significant effect of time interval ( $F_{1,166}=51.31,P<0.001$ , type 3 ANOVA) and inclusion criteria ( $F_{2,166}=8.02,P<0.001$ , type 3 ANOVA). However, when we excluded the “always active GCaMP cells” group, then there was no longer a significant effect between the two remaining inclusion criteria ( $F_{1,109}=0.046,P=0.830$ , type 3 ANOVA), while the effect of time interval remained significant ( $F_{1,109}=33.09,P<0.001$ , type 3 ANOVA). This suggests that the population vector (PV) correlation was not significantly different regardless of whether we registered the GCaMP cells with dTomato landmarks and included only the stable population. The numerically elevated PV correlations when including only always active GCaMP cells were again expected because, by including only active cells, we essentially eliminated the zero terms introduced by inactive cells during correlation calculation. Consistent with previous findings, these results suggest that the hippocampal spatial representation changes over time, even when instability in field of view was accounted for. However, without registering GCaMP cells with the dTomato channel, one cannot assume that cells not present in the field of view were inactive. Therefore, without the dTomato channel, only the active cells expressing GCaMP can be included in the PV correlation analysis, which can result in an overestimation of PV correlations.

In summary, we found that the hippocampal neural ensembles changed over time in terms of both the population of cells active and the spatial coding of the environment. This finding is consistent with previous reports and holds true even if we account for instability in imaging field of view using the dTomato channel, although a more accurate rate of change can be obtained using the dTomato channel.

## DISCUSSION

### Dual-channel miniscope

Here, we presented a dual-channel miniscope that can image two fluorophores in behaving animals. Our design includes a mechanism to calibrate the focal plane of the two channels and is lightweight enough to be carried by adult mice with minimal impact on behavior. We have validated our design at the bench and in vivo in freely behaving animals performing a spatial navigation task. We found that we can image the same field of view across days and the same focal plane across the two channels with minimal cross-talk, while the animals freely behave, wearing the dual-channel miniscope. Furthermore, we demonstrated a use case where we take advantage of an additional static channel to track the same population of neurons even when some neurons were inactive in some recording sessions. We used the dual-channel miniscope to show that the hippocampal spatial representation changes over time, consistent with previous findings. We were able to account for instability in field of view, providing a more credible and accurate estimation of rate of change in the hippocampal neural ensemble.

### Comparison with alternative designs

Our dual-channel miniscope uses two CMOS sensors to achieve simultaneous dual-channel imaging. Other works have used one CMOS sensor combined with dual–band-pass filters/dichroic and two excitation LEDs (*30*), where one of the LEDs turns on at every other frame and illuminates the sample at one of the two excitation wavelengths, resulting in a multiplexed imaging signal at half the frame rate. The benefit of the single–CMOS sensor design is reduced weight and dimensions. Compared to the single–CMOS sensor design (*30*), our design allows us to separate the channels into two independent light paths and use two single–band-pass emission filters. Because all the filters used in miniscopes are designed for collimated light, they often suffer from cross-talk due to imperfectly collimated light. The extent of cross-talk is often much more severe for dual–band-pass filters compared to single–band-pass ones. Hence, using two single–band-pass emission filters in our design provides better filtering quality and minimizes cross-talk between the two channels. The other benefit of having two CMOS sensors is that the relative positions of the two sensors can be easily adjusted to correct for any displacements in the focal plane potentially caused by chromatic aberrations. Further, full frame rates supported by the image sensors can be used because there is no need to multiplex the imaging data from the two sensors. The cons of using two CMOS sensors are increased physical dimensions and substantial increase in weight. However, we have validated our scope in behaving animals, and our results suggest that, after a period of training, mice can perform normally in a spatial navigation task.

### Limitations and future directions

Our dual-channel miniscope uses an excitation LED with a single-peak wavelength, which was sufficient to excite both GCaMP and static dTomato markers. Here we have only demonstrated one use case for our dual-channel miniscope: using an additional static channel to image a constitutively active dTomato fluorophore (together with GCaMP). This allowed us to account for the instability in imaging field of view and track the same population of neurons longitudinally, even when some neurons were not always active. However, our design can be modified for additional applications. For example, imaging a cell-type–specific or activity-dependent marker together with calcium activity can reveal the different activity patterns for neurons with different functions. Furthermore, having two channels opens up the possibility of fluorescence resonance energy transfer (FRET) microscopy using a miniscope. Cell-based neurotransmitter fluorescent engineered reporters (CNiFERs) that emit FRET signals have recently been developed to detect the release of neurotransmitters (*31*–*33*). Our dual-channel miniscope can be modified to image numerous FRET-based CNiFERs to study the dynamics of neurotransmitters. Last, a researcher might image two dynamic fluorescence signals across the two channels, e.g., using GCaMP and RCaMP (a red-shifted calcium indicator) to mark excitatory and inhibitory neurons, respectively, to study the distinct population dynamics. One can also image GCaMP signals from neurons together with dynamics of neurotransmitter release using genetically encoded sensors (*34*–*37*). To image these different combinations of fluorophores, usually either a dual-wavelength LED or two different LEDs are required. In both cases, two independent LED drivers need to be integrated on the printed circuit board (PCB), which is feasible because there is extra space on the PCB in our current prototype and the power drawn from additional LEDs is relatively small. Hence, the next step in future development of our dual-channel miniscope is to enable two excitation wavelengths, which greatly expand the applicability of our dual-channel miniscope.

The dual-channel miniscope weighs 4.8 g, which can be carried by mice but requires a much longer period of training time comparing to single-channel miniscopes. The fact that two coax cables are required for the current dual-channel miniscope prohibits the use of typical commutators, which exacerbates the tether of animals and contributes to the longer training time. To decrease the overall weight of our dual-channel scope and provide compatibility with typical commutators, the PCBs could be designed so that the image sensors and other components are integrated more compactly on a single PCB. Now, the PCBs used in our dual-channel miniscope are two independent PCBs from Miniscope v4 intended to serve as a proof of concept, which means that many integrated circuits (IC) are duplicated and add unnecessary weight to the miniscope. In particular, two serializers on the two separate PCBs necessitate two separate coax cables. Future iterations could include a one-piece PCB that hosts two excitation LEDs and two image sensors, with only one copy of the rest of the components including the electro-wetting lens driver, microcontroller, and serializer. Hence, future development of the dual-channel miniscope can further reduce the weight and provide more flexibility in experimental setups.

To date, we have only validated the dual-channel miniscope with a limited number of experiments. More validations are needed to ensure that our dual-channel miniscope is suitable for broader applications. For example, to fully validate that we can image the same focal plane in behaving animals, we need to express two static markers with different emission wavelengths in the same population of neurons. A high degree of overlap in this case would indicate successful imaging of the same population of neurons across two channels, although this too could be subject to other confounds such as expression-level differences as mentioned. Furthermore, we could image two dynamic calcium indicators (for example, GCaMP and a red-shifted calcium indicator such as RCaMP or jRGECO) in the same field of view with our dual-channel miniscope. We could image the two calcium indicators in the same population of neurons and calculate the correlation of temporal dynamics of the matching cells across the two channels. A high degree of correlation would further confirm that we can image the same focal plane and that the signals are not contaminated by out-of-focus fluorescence, provided that the effect of cross-talk can be ruled out. Additionally, we could image two calcium indicators in different, nonoverlapping subsets of neurons and again investigate the correlation between temporal signals at matching locations in the field of view across two channels to evaluate cross-talk. On the basis of our validation of limited cross-talk between the two channels, we expect no significant correlation of the temporal signals. Still, this is an important validation to confirm that we can image two dynamic signals simultaneously with minimal cross-talk.

In conclusion, we have demonstrated that our dual-channel miniscope is capable of imaging two fluorescence wavelengths in behaving animals. We believe that our dual-channel miniscope has many applications and a substantial impact on modern neuroscience research.

## MATERIALS AND METHODS

### Surgery and imaging experiment

All experimental protocols were approved by the Institutional Animal Care and Use Committee of the Icahn School of Medicine at Mount Sinai (IACUC-2017-0361) in accordance with the US National Institutes of Health (NIH) guidelines. Ten adult male C57BL/6J mice were used during the validation imaging experiment. AAV1-hSyn-GCaMP6f-P2A-nls-dTomato virus [titer 2 × 10^13^ genome copies (GC)/ml] that co-expresses GCaMP and nucleus-localized static dTomato signals was purchased from Addgene, which was a gift from J. Ting (Addgene, viral prep no. 51085-AAV1; http://n2t.net/addgene:51085; RRID:Addgene_51085). For all surgeries, mice were anesthetized with 1.2 to 1.5% isoflurane and head fixed onto a stereotax (David Kopf Instruments). Lidocaine (2%, Akorn) was injected to the neck area as an analgesic. All mice underwent virus injection and lens implantation stereotaxic surgeries on the same day. First, mice were unilaterally injected with 500 nl of AAV1-hSyn-GCaMP6f-P2A-nls-dTomato virus at 2 nl/s in the dorsal CA1 (−2.0 mm anteroposterior relative to bregma, +1.8 mm mediolateral from bregma, and 1.6 mm ventral from the skull surface) using a Nanoject microinjector (Drummond Scientific). Next, mice underwent a GRIN lens implantation surgery. A craniotomy at 1 mm in diameter was performed above the viral injection site. The cortical tissue above the corpus callosum was carefully aspirated using 27- and 30-gauge blunt needles while buffered artificial cerebrospinal fluid [135 mM NaCl, 5 mM KCl, 5 mM Hepes, 2.4 mM CaCl_2_, and 2.1 mM MgCl_2_ (pH 7.4)] was constantly applied throughout the aspiration to prevent desiccation of the tissue. The aspiration ceased after partial removal of the corpus callosum and full termination of bleeding, at which point a GRIN lens (1-mm diameter, 4.0-mm length, 0.5 pitch, Inscopix) was stereotaxically lowered to the targeted implant site (−1.35 mm dorsoventral from the bregma). Cyanoacrylate glue and dental cement were used to seal and cover the exposed skull, and Kwik-Sil (World Precision Instruments, catalog no. 600022) covered the exposed GRIN lens. A final layer of cement was then applied above the Kwik-Sil to protect the lens from group housing. Subcutaneous saline injections were administered at the end of each surgery to prevent dehydration. Carprofen (5 mg/kg), dexamethasone (0.2 mg/kg), and ampicillin (20 mg/kg) were administered during surgery and for 7 days after surgery together. Animals were anesthetized again 3 weeks later, and the dual-channel miniscope with an aluminum baseplate attached was placed on top of the GRIN lens. After searching the field of view for in-focus cells under the dTomato channel, the baseplate was cemented into place, and the miniscope was unlocked and detached from the baseplate. A plastic cap was locked into the baseplate to prevent debris buildup.

During the imaging experiment, mice were water restricted by replacing the water supply with citric acid (4 mg/ml) (*38*). All mice maintained 80 to 85% of their original body weights throughout the imaging experiment. Mice were habituated to run on a 1-m linear track to retrieve water rewards at both ends for 2 weeks before the imaging experiment. Each visit to the two ends of the track was considered one trial. Mice wore custom-made dummy miniscopes with increasing size and weights through the course of habituation until all mice could run at least 10 trials on the linear track within 15 min wearing the dual-channel miniscope with cable attached. The imaging experiment consisted of seven 15-min imaging sessions with a 2-day interval, spanning a total of 13 days. The behavior video was collected with an off-the-shelf webcam (ELP-USBFHD01M-L180, Amazon). The imaging data were collected with standard Miniscope v4 data acquisition hardware and software (https://github.com/Aharoni-Lab/Miniscope-DAQ-QT-Software).

To compare the running speed of mice wearing the dual-channel miniscope with mice wearing a traditional Miniscope v4, we collected data from another group of mice that underwent the same surgical and experimental procedures. This group of mice was trained to collect water rewards on the same 1-m-long linear track while wearing single-channel Miniscope v4. To estimate the animals’ running speed, we calculated the gradient of position change (in pixels) for each frame and then scaled the value to physical units (centimeters per second) based on the track length and video frame rate. We estimated running speed as the 95th percentile of locomotion speed across all frames in a session to avoid biasing results with acceleration or deceleration.

### Additional behavioral validation with the Restaurant Row task

To investigate the behavioral impact of dual-channel miniscope in a more cognitively complex setting, we investigated behavior of mice wearing single-channel and dual-channel miniscope in a neuroeconomic task called Restaurant Row (*39*). Briefly, Restaurant Row is a neuroeconomic foraging task. In this complex decision-making task, mice must forage for their primary source of food while on limited daily time budget in a closed-economy system by navigating a maze with four uniquely flavored and contextualized feeding sites or “restaurants.” Mice learn to associate the pitch of a tone with reward cost in the form of a delay required to obtain food. Mice are free to choose in a self-paced manner how to invest time in competing actions. Mice decided whether to skip offers presented in a spatially segregated offer zone and proceed to the next restaurant or to accept and then separately wait for cued delays in a distinct wait zone. Further details of the Restaurant Row task were previously described (*39*).

To compare the behavioral performance, mice previously trained in the Restaurant Row task were tested while wearing single-channel miniscope in the first day and then tested wearing dual-channel miniscope in the following day. The total distance traveled, the total number of valid laps (defined as number of laps mice ran in the correct order), and the total amount of rewards earned were compared across the 2 days.

### Data analysis

Animal positions were tracked with ezTrack (*40*, *41*) (https://github.com/denisecailab/ezTrack), and the calcium activity from the GCaMP channel was processed using Minian (*42*) (https://github.com/denisecailab/minian). The static dTomato channel was processed using a modified pipeline based on Minian, which detected cells by locating local maxima, and then extracted spatial footprints by detecting round features around the local maxima. For the hippocampal population analysis (Fig. 7), we excluded one animal (m30) because one recording session was missing for that animal. All downstream analyses were performed using in-house Python scripts that are available at https://github.com/denisecailab/Miniscope_2s-validation. Briefly, after extracting the spatial footprints of cells from both dTomato and GCaMP channels, we extracted denoised calcium activity and deconvolved signal using Minian from the GCaMP channel. To benchmark the cross-talk between the two channels, we extracted the equivalent of temporal activity for each cell from the dTomato channel by projecting raw pixel fluorescence values onto the spatial footprints. Next, we registered cells across recording sessions using a centroid-distance–based registration algorithm in Minian. We registered cells using either the dTomato channel or the GCaMP channel. Additionally, we registered each GCaMP cell to a dTomato cell within each session. We then generated a cross-sessional registration mapping for GCaMP cells using the cross-sessional mapping of their corresponding dTomato cells. In this way, the dTomato cells serve as stable landmarks across recording sessions even when the cell was not active and not detected in the GCaMP channel in particular sessions.

Once the cells are registered, we were able to calculate the proportion of cells corresponding to different combination of active sessions (fig. S4). Although intuitive, such calculation suffers from bias caused by combinatorial effects when grouping cells by number of active session. To account for this effect, we defined a per-session probability of cells tracked through different sessions (Fig. 7), using the equation

$$p_{m}=\frac{1}{k}\sum_{i=1}^{k}\frac{N_{\mathrm{mi}}}{N_{i}}$$

where $p_{m}$ is the proportion of cells active across m recording sessions, k is the total number of recording sessions (i.e., k=7 when looking across the whole experiment), $N_{i}$ is the total number of cells detected in session i , and $N_{\mathrm{mi}}$ is the number of cells in session i that are also tracked across exactly m sessions. In other words, this calculation estimates the probability of finding a cell being tracked through a different number of sessions on a per-session basis. Similarly, we can derive the reactivation rate as a special case where we were only looking at a pair of sessions and we calculated probability of finding a cell that is active in both sessions in either session, in other words, m=2 and k=2 . Expanding the summation and explicitly labeling the two sessions as A and B , we have

$$p_{\text{AB}}=\frac{1}{2}\left(\frac{N_{2}}{N_{A}}+\frac{N_{2}}{N_{B}}\right)$$

where $p_{\mathrm{AB}}$ is the reactivation rate regarding sessions A and B ; $N_{A}$ and $N_{B}$ are the total number of cells in sessions A and B , respectively; and $N_{2}$ is the number of cells active in both sessions.

To investigate the hippocampal spatial map, we classified behavior episodes into idle, running left, and running right using a speed threshold of 3 cm/s. We then linearized the position of animals along the track and mapped the positions to the range of 0 to 100. To account for directionality of hippocampal place cells, we mapped the position independently when the animals were running left or right, resulting in a total range of 0 to 200. We used kernel density estimation to estimate both the spatial firing activity from the deconvolved signal and the occupancy from animal positions. A cosine kernel with a bandwidth of 5 cm was used for both spatial firing activity and occupancy. To estimate occupancy, the position of the animal at each frame when the animal was not idle was used to directly estimate the density of the positions (occupancy). To estimate spatial firing activity for each cell, the position of an animal at frames when there was a calcium event (i.e., nonzero value in deconvolved signal) was used and weighted according to the amplitude of the deconvolved signal to estimate the density of firing activity as a function of position. After kernel density estimation, we evenly sampled 200 spatial bins from range of 0 to 200 (including both running directions), equivalent to a resolution of 1 cm/bin. We used the discretely sampled spatial firing activity and occupancy for all further calculation and plotting. To calculate spatial information, we used the equation

$$I=\sum_{i=1}^{N}p_{i}\frac{\lambda_{i}}{\bar{\lambda }}{\text{log}}_{2}\frac{\lambda_{i}}{\bar{\lambda }}$$

where N=200 is the total number of spatial bins, $p_{i}$ is the occupancy at spatial bin i , $\lambda_{i}$ is the spatial firing activity at spatial bin i , and $\bar{\lambda }$ is the mean firing activity across the whole recording session. Next, we calculated normalized spatial firing activity by dividing the spatial firing activity at each spatial bin by the corresponding occupancy. Then, to calculate spatial stability within each recording session, we computed the mean Pearson correlation of normalized spatial firing activity across odd and even trials as well as first and second halves of trials. To calculate spatial stability across recording sessions, we computed the mean Pearson correlation of normalized spatial firing activity for the same registered cell across all combinations of sessions. To compute the activity level for each cell, we took the mean activity across time and computed a quantile of mean activity relative to all other cells in the session. To classify whether a cell is a place cell, we circularly shuffled the deconvolved signal for each cell 500 times. The same calculation was repeated to compute the spatial information and stability for these shuffled activities and to build a null distribution of spatial information and stability for each cell. We classified a cell as a place cell if its spatial stability and spatial information were greater than 95th percentile of the corresponding null distribution. In other words, a place cell was defined as a cell with both significant spatial information and significant spatial stability ( P<0.05 compared to chance). To compute PV correlations between a pair of sessions, we built the PV for each spatial bin from the spatial firing activity across all place cells. For cells in the GCaMP channel, we handled inactive cells in two different ways when building the PV: Either the spatial firing activity of inactive cells was set to zero or the inactive cells were excluded from the PV (leaving only the place cells that were active in both sessions). Then, we computed the Pearson correlation of the PVs corresponding to the same spatial bin, and we used the mean correlation across all spatial bins as the PV correlation between a pair of sessions.

## References

[1] D. Aharoni, B. S. Khakh, A. J. Silva, P. Golshani, All the light that we can see: A new era in miniaturized microscopy. Nat. Methods 16, 11–13 (2018). 10.1038/s41592-018-0266-x PMC8320687 30573833

[2] D. Aharoni, T. M. Hoogland, Circuit investigations with open-source miniaturized microscopes: Past, present and future. Front. Cell. Neurosci. 13, 141 (2019). 31024265 10.3389/fncel.2019.00141 PMC6461004

[3] K. Chen, Z. Tian, L. Kong, Advances of optical miniscopes for in vivo imaging of neural activity in freely moving animals. Front. Neurosci. 16, 994079 (2022). 36161177 10.3389/fnins.2022.994079 PMC9490007

[4] Y. Ziv, L. D. Burns, E. D. Cocker, E. O. Hamel, K. K. Ghosh, L. J. Kitch, A. E. Gamal, M. J. Schnitzer, Long-term dynamics of CA1 hippocampal place codes. Nat. Neurosci. 16, 264–266 (2013). 23396101 10.1038/nn.3329 PMC3784308

[5] D. J. Cai, D. Aharoni, T. Shuman, J. Shobe, J. Biane, W. Song, B. Wei, M. Veshkini, M. La-Vu, J. Lou, S. E. Flores, I. Kim, Y. Sano, M. Zhou, K. Baumgaertel, A. Lavi, M. Kamata, M. Tuszynski, M. Mayford, P. Golshani, A. J. Silva, A shared neural ensemble links distinct contextual memories encoded close in time. Nature 534, 115–118 (2016). 27251287 10.1038/nature17955 PMC5063500

[6] T. Okuyama, T. Kitamura, D. S. Roy, S. Itohara, S. Tonegawa, Ventral CA1 neurons store social memory. Science 353, 1536–1541 (2016). 27708103 10.1126/science.aaf7003 PMC5493325

[7] A. Klaus, G. J. Martins, V. B. Paixao, P. Zhou, L. Paninski, R. M. Costa, The spatiotemporal organization of the striatum encodes action space. Neuron 95, 1171–1180.e7 (2017). 28858619 10.1016/j.neuron.2017.08.015 PMC5584673

[8] K.-S. Chen, M. Xu, Z. Zhang, W.-C. Chang, T. Gaj, D. V. Schaffer, Y. Dan, A hypothalamic switch for REM and non-REs sleep. Neuron 97, 1168–1176.e4 (2018). 29478915 10.1016/j.neuron.2018.02.005

[9] J. C. Jimenez, K. Su, A. R. Goldberg, V. M. Luna, J. S. Biane, G. Ordek, P. Zhou, S. K. Ong, M. A. Wright, L. Zweifel, L. Paninski, R. Hen, M. A. Kheirbek, Anxiety cells in a hippocampal-hypothalamic circuit. Neuron 97, 670–683.e6 (2018). 29397273 10.1016/j.neuron.2018.01.016 PMC5877404

[10] L. Kingsbury, S. Huang, J. Wang, K. Gu, P. Golshani, Y. E. Wu, W. Hong, Correlated neural activity and encoding of behavior across brains of socially interacting animals. Cell 178, 429–446.e16 (2019). 31230711 10.1016/j.cell.2019.05.022 PMC6625832

[11] T. Shuman, D. Aharoni, D. J. Cai, C. R. Lee, S. Chavlis, L. Page-Harley, L. M. Vetere, Y. Feng, C. Y. Yang, I. Mollinedo-Gajate, L. Chen, Z. T. Pennington, J. Taxidis, S. E. Flores, K. Cheng, M. Javaherian, C. C. Kaba, N. Rao, M. La-Vu, I. Pandi, M. Shtrahman, K. I. Bakhurin, S. C. Masmanidis, B. S. Khakh, P. Poirazi, A. J. Silva, P. Golshani, Breakdown of spatial coding and interneuron synchronization in epileptic mice. Nat. Neurosci. 23, 229–238 (2020). 31907437 10.1038/s41593-019-0559-0 PMC7259114

[12] J. E. Markowitz, W. A. Liberti, G. Guitchounts, T. Velho, C. Lois, T. J. Gardner, Mesoscopic patterns of neural activity support songbird cortical sequences. PLOS Biol. 13, e1002158 (2015). 26039895 10.1371/journal.pbio.1002158 PMC4454690

[13] W. A. Liberti III, T. A. Schmid, A. Forli, M. Snyder, M. M. Yartsev, A stable hippocampal code in freely flying bats. Nature 604, 98–103 (2022). 35355012 10.1038/s41586-022-04560-0 PMC10212506

[14] A. D. Jacob, A. I. Ramsaran, A. J. Mocle, L. M. Tran, C. Yan, P. W. Frankland, S. A. Josselyn, A compact head-mounted endoscope for in vivo calcium imaging in freely behaving mice. Curr. Protoc. Neurosci. 84, e51 (2018). 29944206 10.1002/cpns.51

[15] A. de Groot, B. J. van den Boom, R. M. van Genderen, J. Coppens, J. van Veldhuijzen, J. Bos, H. Hoedemaker, M. Negrello, I. Willuhn, C. I. De Zeeuw, T. M. Hoogland, NINscope, a versatile miniscope for multi-region circuit investigations. eLife 9, e49987 (2020). 31934857 10.7554/eLife.49987 PMC6989121

[16] B. B. Scott, S. Y. Thiberge, C. Guo, D. G. R. Tervo, C. D. Brody, A. Y. Karpova, D. W. Tank, Imaging cortical dynamics in GCaMP transgenic rats with a head-mounted widefield macroscope. Neuron 100, 1045–1058.e5 (2018). 30482694 10.1016/j.neuron.2018.09.050 PMC6283673

[17] J. Juneau, G. Duret, J. P. Chu, A. V. Rodriguez, S. Morozov, D. Aharoni, J. T. Robinson, F. St-Pierre, C. Kemere, MiniFAST: A sensitive and fast miniaturized microscope for in vivoneural recording. bioRxiv 367466 [Preprint] (2020); 10.1101/2020.11.03.367466. PMC12988765 41837229

[18] J. R. Scherrer, G. F. Lynch, J. J. Zhang, M. S. Fee, A novel optical design enabling lightweight and large field-of-view head-mounted microscopes. bioRxiv 458947 [Preprint] (2021); 10.1101/2021.09.03.458947. 36928075

[19] M. L. Rynes, D. A. Surinach, S. Linn, M. Laroque, V. Rajendran, J. Dominguez, O. Hadjistamoulou, Z. S. Navabi, L. Ghanbari, G. W. Johnson, M. Nazari, M. H. Mohajerani, S. B. Kodandaramaiah, Miniaturized head-mounted microscope for whole-cortex mesoscale imaging in freely behaving mice. Nat. Methods 18, 417–425 (2021). 33820987 10.1038/s41592-021-01104-8 PMC8034419

[20] C. Guo, G. J. Blair, M. Sehgal, F. N. Sangiuliano Jimka, A. Bellafard, A. J. Silva, P. Golshani, M. A. Basso, H. T. Blair, D. Aharoni, Miniscope-LFOV: A large-field-of-view, single-cell-resolution, miniature microscope for wired and wire-free imaging of neural dynamics in freely behaving animals. Sci. Adv. 9, eadg3918 (2023). 37083539 10.1126/sciadv.adg3918 PMC10121160

[21] W. A. Liberti III, L. N. Perkins, D. P. Leman, T. J. Gardner, An open source, wireless capable miniature microscope system. J. Neural Eng. 14, 045001 (2017). 28514229 10.1088/1741-2552/aa6806 PMC5955387

[22] O. Skocek, T. Nöbauer, L. Weilguny, F. Martínez Traub, C. N. Xia, M. I. Molodtsov, A. Grama, M. Yamagata, D. Aharoni, D. D. Cox, P. Golshani, A. Vaziri, High-speed volumetric imaging of neuronal activity in freely moving rodents. Nat. Methods 15, 429–432 (2018). 29736000 10.1038/s41592-018-0008-0 PMC7990085

[23] K. Yanny, N. Antipa, W. Liberti, S. Dehaeck, K. Monakhova, F. L. Liu, K. Shen, R. Ng, L. Waller, Miniscope3D: Optimized single-shot miniature 3D fluorescence microscopy. Light Sci. Appl. 9, 171 (2020). 33082940 10.1038/s41377-020-00403-7 PMC7532148

[24] Y. Xue, I. G. Davison, D. A. Boas, L. Tian, Single-shot 3D wide-field fluorescence imaging with a computational miniature mesoscope. Sci. Adv. 6, eabb7508 (2020). 33087364 10.1126/sciadv.abb7508 PMC7577725

[25] Y. Xue, Q. Yang, G. Hu, K. Guo, L. Tian, Deep-learning-augmented computational miniature mesoscope. Optica 9, 1009 (2022). 36506462 10.1364/optica.464700 PMC9731182

[26] L. Sheintuch, A. Rubin, N. Brande-Eilat, N. Geva, N. Sadeh, O. Pinchasof, Y. Ziv, Tracking the same neurons across multiple days in Ca^2+^ imaging data. Cell Rep. 21, 1102–1115 (2017). 29069591 10.1016/j.celrep.2017.10.013 PMC5670033

[27] A. Rubin, N. Geva, L. Sheintuch, Y. Ziv, Hippocampal ensemble dynamics timestamp events in long-term memory. eLife 4, e12247 (2015). 26682652 10.7554/eLife.12247 PMC4749549

[28] J. O’Keefe, J. Dostrovsky, The hippocampus as a spatial map. Preliminary evidence from unit activity in the freely-moving rat. Brain Res. 34, 171–175 (1971). 5124915 10.1016/0006-8993(71)90358-1

[29] H. Eichenbaum, P. Dudchenko, E. Wood, M. Shapiro, H. Tanila, The hippocampus, memory, and place cells. Neuron 23, 209–226 (1999). 10399928 10.1016/s0896-6273(00)80773-4

[30] V. A. Kveim, L. Salm, T. Ulmer, M. Lahr, S. Kandler, F. Imhof, F. Donato, Divergent recruitment of developmentally defined neuronal ensembles supports memory dynamics. Science 385, eadk0997 (2024). 39146420 10.1126/science.adk0997

[31] E. Lacin, A. Muller, M. Fernando, D. Kleinfeld, P. A. Slesinger, Construction of cell-based neurotransmitter fluorescent engineered reporters (CNiFERs) for optical detection of neurotransmitters in vivo. J. Vis. Exp., 53290 (2016). 27214050 10.3791/53290 PMC4939270

[32] A. Muller, V. Joseph, P. A. Slesinger, D. Kleinfeld, Cell-based reporters reveal in vivo dynamics of dopamine and norepinephrine release in murine cortex. Nat. Methods 11, 1245–1252 (2014). 25344639 10.1038/nmeth.3151 PMC4245316

[33] Q.-T. Nguyen, L. F. Schroeder, M. Mank, A. Muller, P. Taylor, O. Griesbeck, D. Kleinfeld, An in vivo biosensor for neurotransmitter release and in situ receptor activity. Nat. Neurosci. 13, 127–132 (2009). 20010818 10.1038/nn.2469 PMC3992257

[34] T. Patriarchi, J. R. Cho, K. Merten, M. W. Howe, A. Marley, W.-H. Xiong, R. W. Folk, G. J. Broussard, R. Liang, M. J. Jang, H. Zhong, D. Dombeck, M. von Zastrow, A. Nimmerjahn, V. Gradinaru, J. T. Williams, L. Tian, Ultrafast neuronal imaging of dopamine dynamics with designed genetically encoded sensors. Science 360, eaat4422 (2018). 29853555 10.1126/science.aat4422 PMC6287765

[35] F. Sun, J. Zeng, M. Jing, J. Zhou, J. Feng, S. F. Owen, Y. Luo, F. Li, H. Wang, T. Yamaguchi, Z. Yong, Y. Gao, W. Peng, L. Wang, S. Zhang, J. Du, D. Lin, M. Xu, A. C. Kreitzer, G. Cui, Y. Li, A genetically encoded fluorescent sensor enables rapid and specific detection of dopamine in flies, fish, and mice. Cell 174, 481–496.e19 (2018). 30007419 10.1016/j.cell.2018.06.042 PMC6092020

[36] J. Feng, C. Zhang, J. E. Lischinsky, M. Jing, J. Zhou, H. Wang, Y. Zhang, A. Dong, Z. Wu, H. Wu, W. Chen, P. Zhang, J. Zou, S. A. Hires, J. J. Zhu, G. Cui, D. Lin, J. Du, Y. Li, A genetically encoded fluorescent sensor for rapid and specific in vivo detection of norepinephrine. Neuron 102, 745–761.e8 (2019). 30922875 10.1016/j.neuron.2019.02.037 PMC6533151

[37] J. Wan, W. Peng, X. Li, T. Qian, K. Song, J. Zeng, F. Deng, S. Hao, J. Feng, P. Zhang, Y. Zhang, J. Zou, S. Pan, M. Shin, B. J. Venton, J. J. Zhu, M. Jing, M. Xu, Y. Li, A genetically encoded sensor for measuring serotonin dynamics. Nat. Neurosci. 24, 746–752 (2021). 33821000 10.1038/s41593-021-00823-7 PMC8544647

[38] A. E. Urai, V. Aguillon-Rodriguez, I. C. Laranjeira, F. Cazettes, Z. F. Mainen, A. K. Churchland, Citric acid water as an alternative to water restriction for high-yield mouse behavior. ENeuro 8, ENEURO.0230-20.2020 (2021). 10.1523/ENEURO.0230-20.2020 PMC7890523 33431508

[39] C. A. Nwakama, R. Durand-de Cuttoli, Z. M. Oketokoun, S. O. Brown, J. E. Haller, A. Méndez, M. Jodeiri Farshbaf, Y. Z. Cho, S. Ahmed, S. Leng, J. L. Ables, B. M. Sweis, Neuroeconomically dissociable forms of mental accounting are altered in a mouse model of diabetes. Commun. Biol. 8, 102 (2025). 39838110 10.1038/s42003-025-07500-6 PMC11751097

[40] Z. T. Pennington, Z. Dong, Y. Feng, L. M. Vetere, L. Page-Harley, T. Shuman, D. J. Cai, ezTrack: An open-source video analysis pipeline for the investigation of animal behavior. Sci. Rep. 9, 19979 (2019). 31882950 10.1038/s41598-019-56408-9 PMC6934800

[41] Z. T. Pennington, K. S. Diego, T. R. Francisco, A. R. LaBanca, S. I. Lamsifer, O. Liobimova, T. Shuman, D. J. Cai, ezTrack—A step-by-step guide to behavior tracking. Curr. Protoc. 1, e255 (2021). 34610215 10.1002/cpz1.255 PMC8500532

[42] Z. Dong, W. Mau, Y. Feng, Z. T. Pennington, L. Chen, Y. Zaki, K. Rajan, T. Shuman, D. Aharoni, D. J. Cai, Minian, an open-source miniscope analysis pipeline. eLife 11, e70661 (2022). 35642786 10.7554/eLife.70661 PMC9205633

[43] S. Malvaut, A. Marymonchyk, A. Gengatharan, A. Saghatelyan, Live imaging of adult neural stem cells in freely behaving mice using mini-endoscopes. STAR Protocols 2, 100596 (2021). 34169290 10.1016/j.xpro.2021.100596 PMC8209737

[44] W. Zong, R. Wu, S. Chen, J. Wu, H. Wang, Z. Zhao, G. Chen, R. Tu, D. Wu, Y. Hu, Y. Xu, Y. Wang, Z. Duan, H. Wu, Y. Zhang, J. Zhang, A. Wang, L. Chen, H. Cheng, Miniature two-photon microscopy for enlarged field-of-view, multi-plane and long-term brain imaging. Nat. Methods 18, 46–49 (2021). 33408404 10.1038/s41592-020-01024-z

[45] B. A. Flusberg, A. Nimmerjahn, E. D. Cocker, E. A. Mukamel, R. P. J. Barretto, T. H. Ko, L. D. Burns, J. C. Jung, M. J. Schnitzer, High-speed, miniaturized fluorescence microscopy in freely moving mice. Nat. Methods 5, 935–938 (2008). 18836457 10.1038/nmeth.1256 PMC2828344

[46] W. Zong, H. A. Obenhaus, E. R. Skytøen, H. Eneqvist, N. L. de Jong, R. Vale, M. R. Jorge, M.-B. Moser, E. I. Moser, Large-scale two-photon calcium imaging in freely moving mice. Cell 185, 1240–1256.e30 (2022). 35305313 10.1016/j.cell.2022.02.017 PMC8970296

[47] B. A. Madruga, C. C. Dorian, M. Sehgal, A. J. Silva, M. Shtrahman, D. Aharoni, P. Golshani, Open-source, high performance miniature multiphoton microscopy systems for freely behaving animals. bioRxiv 586663 [Preprint] (2024); 10.1101/2021.09.03.458947. PMC12318034 40753159

[48] O. D. Supekar, A. Sias, S. R. Hansen, G. Martinez, G. C. Peet, X. Peng, V. M. Bright, E. G. Hughes, D. Restrepo, D. P. Shepherd, C. G. Welle, J. T. Gopinath, E. A. Gibson, Miniature structured illumination microscope for in vivo 3D imaging of brain structures with optical sectioning. Biomed. Opt. Express 13, 2530–2541 (2022). 35519247 10.1364/BOE.449533 PMC9045919

