# Supplementary material for: Simultaneous two-color imaging with a dual-channel miniscope in freely behaving mice
Source: Sci Adv. 2025 Jul 2;11(27):eadr6470. doi: 10.1126/sciadv.adr6470 (PMC12219470; doi:10.1126/sciadv.adr6470)
Supplement: Supplementary file 1 — Table S1 Figs. S1 to S5 Legend for movie S1 [file sciadv.adr6470_sm.pdf]

Science Advances
AAAS

Supplementary Materials for

Simultaneous two-color imaging with a dual-channel miniscope in freely behaving mice

Zhe Dong *et al.*

Corresponding author: Denise J. Cai, denise.cai@mssm.edu

Sci. Adv. **11**, eadr6470 (2025)
DOI: 10.1126/sciadv.adr6470

The PDF file includes:

Table S1
Figs. S1 to S5
Legend for movie S1

Other Supplementary Material for this manuscript includes the following:

Movie S1

Supplementary Materials

Table S1: ***Bill of materials for dual-channel Miniscope.*** *The name, vendor, part number and description are shown for each part.*

Name	Vendor	Part number	Description
Excitation filter	Chroma	ZET488/561x	488nm and 561 nm dual-band pass
Excitation dichroic mirror	Chroma	ZT488/561rpc	Reflects wavelengths between 482-493nm and 555-566nm
Emission dichroic mirror	Chroma	T556lpxr	556nm high-pass
Side emission filter	Chroma	ET525/50m	525nm band pass
Top emission filter	Chroma	ET600/50m	600nm band pass
LED	Lumileds	LXZ1-PB01	high-power LED with 460-480nm dominant wavelength
Objective achromatic lens	Edmund Optics	45-089	3mm diameter 6mm FL achromat
Achromatic lens	Edmund Optics	63-691	4mm diameter 10mm FL achromat
Electro-wetting lens	Edmund Optics	34-282	2.5mm CA A-25H0 Corning Varioptic Variable Focus Liquid Lens

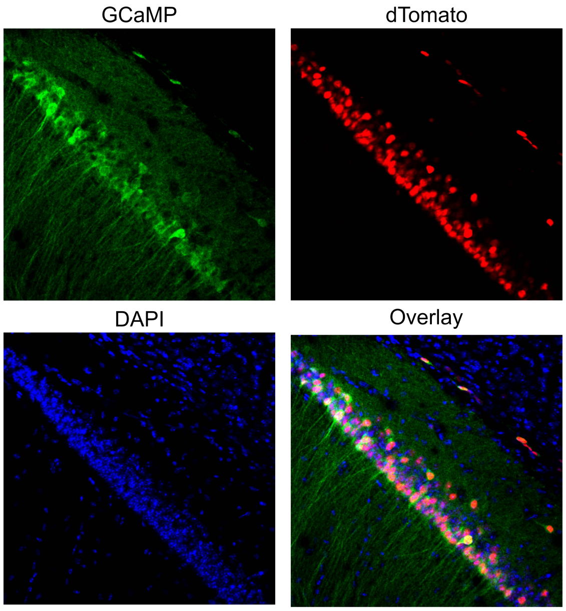

Figure S1: ***Confocal validation of virus expression.*** *Expression profile of AAV1-hSyn-GCaMP6f-P2A-nls-dTomato virus in hippocampus CA1 is shown.*

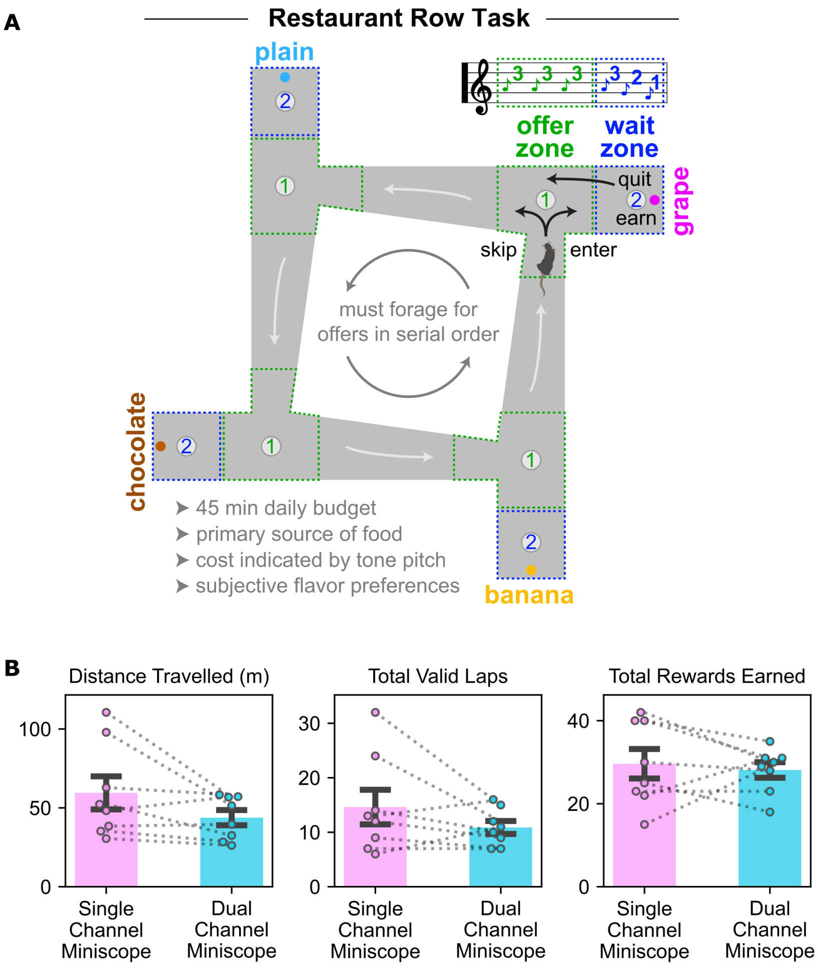

Figure S2: ***Behavioral validation in the Restaurant Row task. (A)*** *Schematic of the Restaurant Row task. Mice were required to run in a counterclockwise direction encountering offers for different flavors at each "restaurant" in serial order. Each restaurant, separated by hallways, was divided into a T-shaped "offer zone" choice point and a separate "wait zone" that housed the pellet dispenser. Upon offer zone entry from the correct heading direction, a tone sounded whose pitch indicated the delay mice would have to wait if accepting the offer by entering the wait zone. Each trial terminated if mice skipped in the offer zone, quit during the countdown in the wait zone, or earned a reward, after which animals were required to proceed to the next restaurant. This schematic was adopted from (39)* ***(B)*** *Behavioral metrics of animals wearing dual-channel and single-channel Miniscope in the Restaurant Row task. The same animals were trained in the task wearing single-channel and dual-channel Miniscope. No significant difference were observed between single-channel and dual-channel Miniscope groups in total distance traveled ($p = 0.092$, paired t-test), total valid laps (defined as number of laps animal ran in the counterclockwise direction, $p = 0.195$, paired t-test), and total rewards earned ($p = 0.650$, paired t-test).*

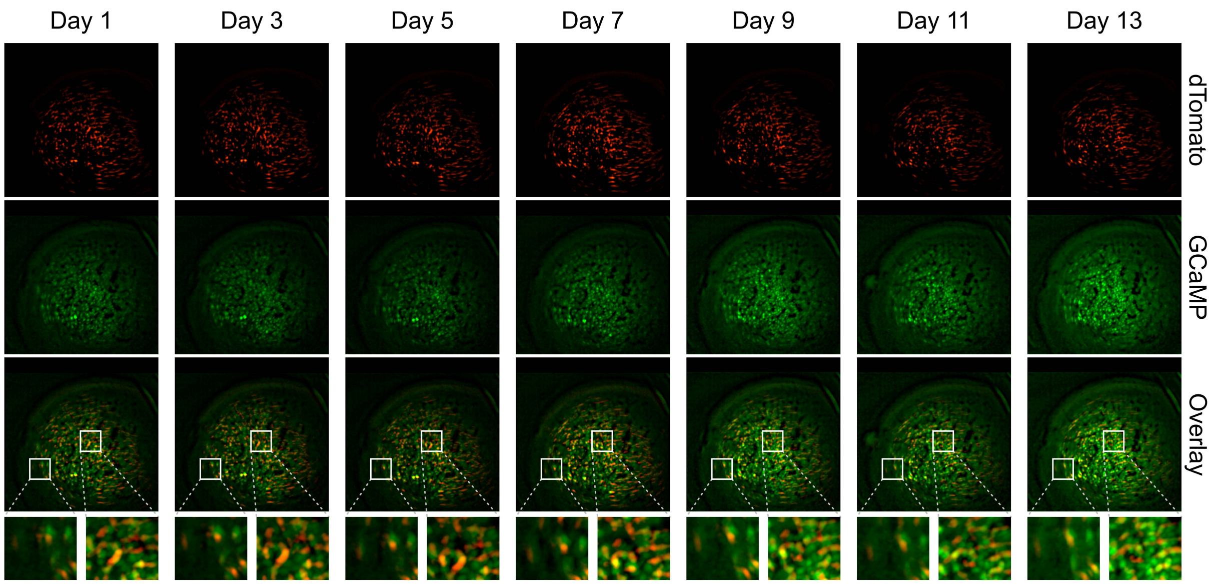

Figure S3: ***Representative field of view from a single animal throughout the experiment.*** *Maximum projection of processed imaging data are pseudo-colored and shown for the tdTomato (top row) and GCaMP (middle row) channels and the overlay images (bottom row). The zoomed insets show the overlay at the center and edge of the field of view for each day.*

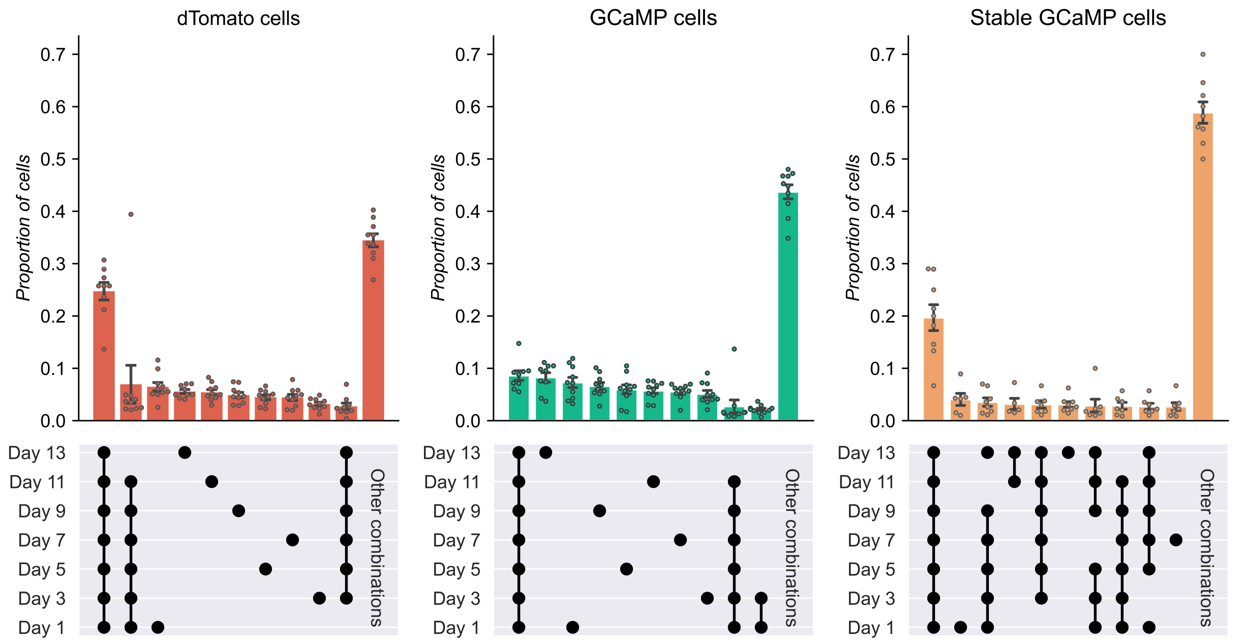

Figure S4: ***Detailed breakdown of cells tracked across different sessions.*** *The proportion of cells tracked across different combinations of sessions are shown for tdTomato (left), GCaMP (middle) and stable GCaMP cells (right). Different combinations of sessions are represented by dots and lines at the bottom of each plot, where a black dot on the corresponding day indicates that cells were active on that day, and lines connecting dots indicate cells were tracked continuously across the corresponding days. For example, the first bar of each plot represent*

the proportion of cells that were tracked across all 7 recording sessions. The combinations are sorted by the corresponding proportion of cells in descending order, and only the top 10 combinations are shown, with the rest grouped together as "Other combinations".

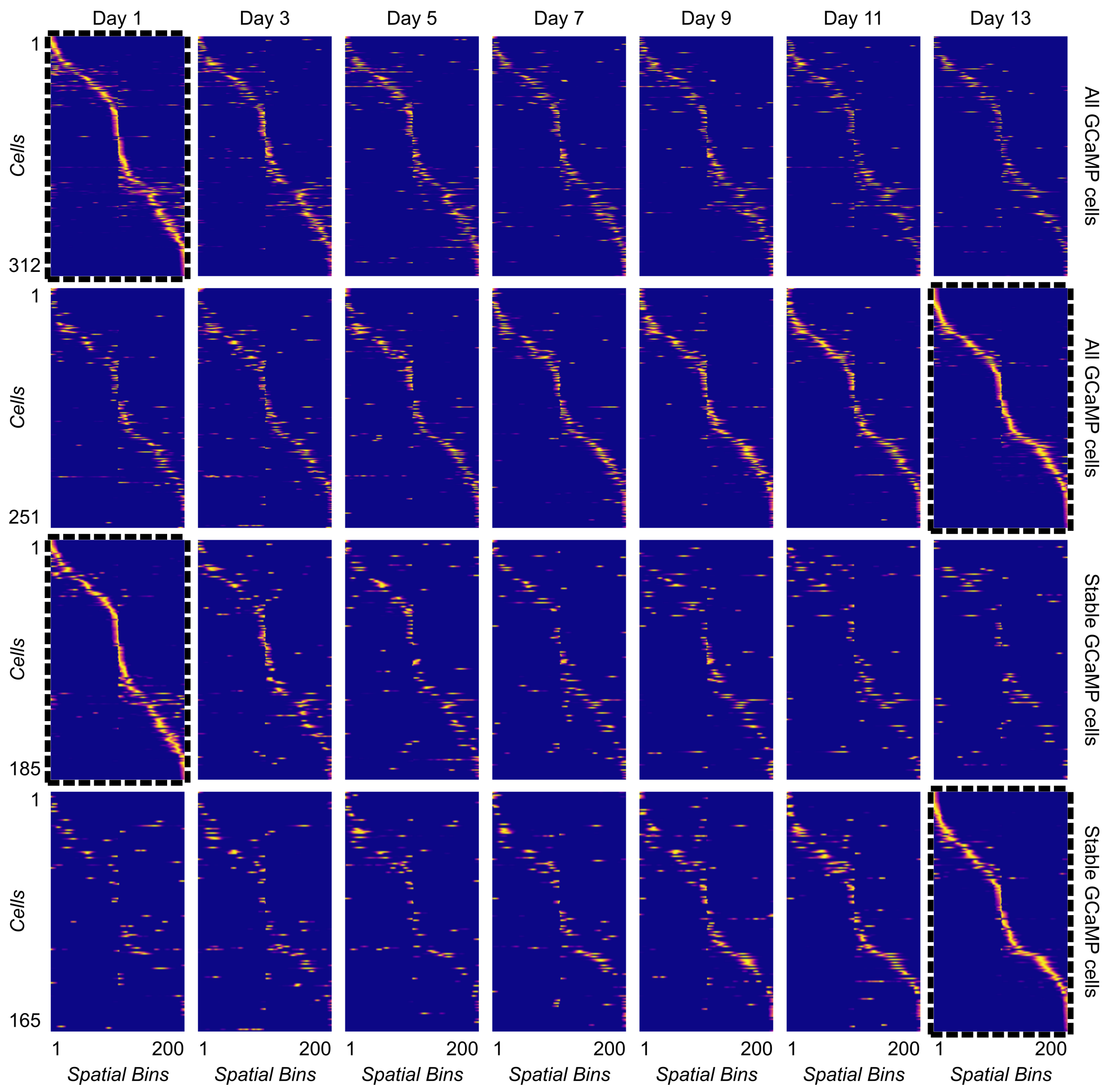

Figure S5: ***Spatial firing patterns of cells tracked across the full experiment.*** *Each row represents a cell tracked throughout the experiment and each column represents a spatial bin. The average firing rate is shown across 200 spatial bins (100 spatial bins for each running direction). First row: all cells in GCaMP channel are included and sorted according to peak firing position in the first day. Second row: same population of cells as first row but cells are sorted according to peak firing position in the last day. Third row: only GCaMP cells whose corresponding tdTomato landmark can be tracked stably across all 7 recording sessions are included. The cells are sorted according to peak firing position in the first day. Fourth row: same*

population of cells as the third row but cells are sorted according to peak firing position in the last session.

Supplementary File S1: **Video of in vivo validation experiment**. The behavioral recording of mouse running on the linear track with dual-channel Miniscope are shown at the top. The corresponding raw Miniscope recording are shown in the middle (left: GCaMP channel, center: tdTomato channel, right: overlap). The corresponding processed signals are shown at the bottom (left: extract calcium signals in GCaMP channel, center: processed signal before cell segmentation in the tdTomato channel, right: overlap).
